# Equity and Transportability of Plasma ATN Phenotypes in a Population-Representative U.S. Aging Cohort

**DOI:** 10.64898/2026.01.31.26344775

**Published:** 2026-02-05

**Authors:** Emmanuel Fle Chea

**Affiliations:** Independent Researcher, University of Minnesota School of Public Health (Alumnus), Minneapolis, MN, USA

**Keywords:** Alzheimer’s disease, plasma biomarkers, ATN framework, health equity, transportability, fairness metrics, structural disadvantage, vascular comorbidity

## Abstract

Plasma biomarkers are transforming Alzheimer’s disease research, yet their performance in diverse, population-representative settings remains largely unknown. Using data from 4,427 adults in the nationally representative Health and Retirement Study, this work evaluates whether amyloid, tau, and neurodegeneration (ATN) biomarkers transport equitably across demographic groups. Population-weighted analyses reveal that tau is the only biomarker showing a stable association with cognition at the population level, whereas amyloid and neurodegeneration markers lose significance after accounting for sampling design. Fairness analyses uncover striking disparities: sensitivity for detecting cognitive impairment is more than twofold higher in White than Black participants, with Black women exhibiting the lowest detection rates. Education further modifies biomarker-cognition relationships, producing paradoxical positive amyloid associations and amplified neurodegeneration effects in structurally disadvantaged groups. These patterns persist after adjustment for vascular comorbidities, indicating that equity gaps arise from more than differential cerebrovascular burden. Together, these findings show that plasma ATN biomarkers do not generalize uniformly across the U.S. population and may systematically underperform in minoritized and socioeconomically disadvantaged groups. The results highlight the need for population-based validation and fairness-aware calibration before deploying blood-based biomarkers in clinical or public health settings.

## INTRODUCTION

Plasma biomarkers for Alzheimer’s disease have been promoted as a major step toward scalable, accessible detection of amyloid, tau, and neurodegeneration.[1,2] By extending the ATN framework beyond CSF and PET imaging, these assays are expected to broaden diagnostic reach and reduce long-standing barriers to participation in dementia research and care. Yet the rapid adoption of plasma biomarkers has outpaced evidence about how well they perform in the populations most likely to benefit from them.

Most validation studies come from highly selected clinical cohorts that differ sharply from the general population in ancestry, education, socioeconomic status, and comorbidity burden. These sampling patterns raise fundamental questions about transportability: whether biomarker thresholds, effect sizes, and predictive performance derived from convenience samples hold in diverse, population-representative settings. At the same time, little is known about whether plasma biomarkers exhibit systematic performance differences across demographic groups, an equity concern with direct implications for screening, diagnosis, and clinical trial enrollment.

A second gap concerns fairness. As biomarker-based risk stratification becomes more common, it is essential to understand whether sensitivity, specificity, and calibration vary across race, ethnicity, sex, or their intersections.[8] Such disparities could amplify existing inequities in dementia detection and access to care. Finally, structural disadvantage, captured here through educational attainment as a proxy for life-course socioeconomic adversity, may modify biomarker-cognition relationships in ways that remain largely unexplored.

The Health and Retirement Study provides a rare opportunity to address these gaps using a nationally representative cohort with plasma biomarker data. In this study, we evaluate the population-level transportability of plasma ATN phenotypes, quantify fairness disparities across demographic subgroups, and test whether structural disadvantage modifies biomarker-cognition associations.[7] Taken together, these analyses provide, to our knowledge, the first population-representative evaluation of equity and transportability in plasma ATN biomarkers and identify the circumstances in which blood-based screening may contribute to either the amplification or reduction of health disparities.

## METHODS

### Study Population

The Health and Retirement Study is a nationally representative longitudinal panel study of U.S. adults aged ≥50 years, initiated in 1992 and conducted biennially [15]. HRS employs multistage probability sampling with oversampling of Black and Hispanic individuals to ensure adequate representation. The study protocol was approved by the University of Michigan Institutional Review Board, and all participants provided informed consent.

We analyzed data from the 2016 HRS Venous Blood Study (VBS), which collected blood samples from 9,934 participants during in-person interviews [16]. Plasma biomarker assays (Aβ42/40 ratio, pTau181, NfL, GFAP) were completed on 4,465 participants. After excluding participants with missing biomarker data (n=38), our final analytic sample comprised 4,427 individuals with complete ATN classification and cognitive assessment (Table 1). Demographic characteristics, educational attainment, and survey weights were obtained from the 2020 HRS Tracker File.

### Plasma Biomarker Measurement and ATN Classification

Plasma biomarkers were measured using Simoa assays for Aβ42/40 ratio, pTau181, NfL, and GFAP.[1,2] All assays were performed at a central laboratory following standardized protocols [16].

ATN classification followed established criteria [3]:

- **A (Amyloid):** Aβ42/40 ratio < 0.063 = positive
- **T (Tau):** pTau181 > 2.5 pg/mL = positive
- **N (Neurodegeneration):** NfL > 20 pg/mL OR GFAP > 100 pg/mL = positive

We classified participants into eight ATN profiles (A−T−N−, A+T−N−, A−T+N−, A−T−N+, A+T+N−, A+T−N+, A−T+N+, A+T+N+) and a simplified four-category scheme:

- **AD pathology:** A+T+ (regardless of N)
- **Amyloid only:** A+T−
- **Suspected non**-**AD pathology:** A−(T+ or N+)
- **Normal biomarkers:** A−T−N−

### Cognitive Assessment

Cognitive function was assessed using the Telephone Interview for Cognitive Status, modified (TICSm), a 27-point scale comprising immediate and delayed recall, serial 7s subtraction, and backward counting [18]. Cognitive impairment classification followed validated criteria [19]:

- **Dementia:** TICSm ≤ 6
- **Cognitive impairment, no dementia (CIND):** TICSm 7–11
- **Normal cognition:** TICSm ≥ 12

We analyzed 2016 cognition scores as the primary outcome, with secondary analyses examining 2018 and 2020 assessments to evaluate longitudinal change.

### Demographic and Socioeconomic Variables

Race/ethnicity was categorized as non-Hispanic White, non-Hispanic Black, Hispanic (any race), or Other. Sex was coded as male or female based on self-report. Age was analyzed continuously and categorized (<65, 65–74, 75–84, ≥85 years). Educational attainment was measured as years of schooling and categorized as: less than high school (<12 years), high school graduate (12 years), some college (13–15 years), or college graduate (≥16 years). For interaction models, we created a binary indicator for low education (<12 years) representing structural disadvantage [13].

Intersectional demographic groups were created by crossing race/ethnicity with sex, yielding four primary strata for analysis: White women, White men, Black women, and Black men. Hispanic and Other racial/ethnic groups were included in overall analyses but excluded from some stratified models due to sample size limitations.

### Vascular Comorbidity Assessment

Vascular comorbidity data were extracted from the 2016 HRS Health module. Self-reported physician-diagnosed conditions included hypertension, diabetes, and stroke. We constructed a vascular comorbidity burden score (range 0–3) by summing these conditions, with a binary indicator for any cardiovascular disease (CVD_Any).

### Survey Weighting and Complex Sampling Design

HRS employs complex multistage probability sampling with stratification and clustering. We utilized person-level survey weights (PVBSWGTR) that adjust for sampling probability, non-response, and post-stratification to Current Population Survey totals [17]. Survey design variables included primary sampling units (SECU) and strata (STRATUM).

All descriptive statistics and regression models were estimated using survey-weighted methods via the R survey package [20] to produce population-representative estimates. Unweighted estimates were computed for comparison to evaluate transportability.

### Statistical Analysis

#### Transportability Framework:

We compared weighted versus unweighted estimates for[7]: (1) ATN prevalence across all eight profiles and the four-category classification; (2) biomarker-cognition associations from multivariable linear regression models adjusting for age, sex, race/ethnicity, and education. Differences between weighted and unweighted estimates quantify selection bias and inform the transportability of results from convenience samples to the U.S. population.

#### Fairness Metrics:

We defined “predicted high risk” as A+T+ (AD pathology) and “actual outcome” as cognitive impairment (CIND or dementia).[8] Standard classification metrics were calculated by race/ethnicity, sex, and intersectional subgroups:

$$TPR(\text{Sensitivity})=\frac{TP}{TP+FN}$$

$$FPR(1-\text{Specificity})=\frac{FP}{FP+TN}$$

$$PPV(\text{Precision})=\frac{TP}{TP+FP}$$

$$NPV=\frac{TN}{TN+FN}$$

$$\text{Accuracy}=\frac{TP+TN}{TP+FP+TN+FN}$$

Disparities were quantified as absolute differences in TPR between demographic groups, with higher values indicating superior sensitivity for detecting true cognitive impairment [11,12].

#### Education and Structural Disadvantage:

We conducted three complementary analyses: (1) education-stratified prevalence of ATN profiles; (2) education-stratified multivariable regression models examining biomarker-cognition associations within each education stratum; (3) interaction models testing biomarker × education interactions on cognitive performance. These analyses evaluate whether structural disadvantage (operationalized as low education) modifies the relationship between plasma biomarkers and cognition [13,14].

#### Race × Biomarker Interactions:

To assess differential biomarker effects across racial/ethnic groups, we fitted multivariable linear regression models with race/ethnicity × biomarker interaction terms:

$$\text{Cognition}=\beta_{0}+\beta_{A}A+\beta_{T}T+\beta_{N}N+\beta_{R}\text{Race}+\beta_{AR}(A\times \text{Race})+\beta_{TR}(T\times \text{Race})+\beta_{NR}(N\times \text{Race})+\gamma X$$

where *X* represents covariates (age, sex, education). Significant interaction terms indicate that the relationships between biomarkers and cognition differ by race/ethnicity [21].

#### Vascular Comorbidity Adjustment:

We fitted models both with and without adjustment for vascular comorbidities (hypertension, diabetes, stroke) to assess whether cerebrovascular burden confounds or modulates biomarker-cognition associations. We also stratified analyses by CVD presence (none vs. any) to test effect modification.

#### Calibration Analysis:

We assessed biomarker calibration by comparing predicted versus observed cognitive impairment rates across risk deciles, stratified by race/ethnicity. Predicted risk was derived from logistic regression models including A, T, and N biomarkers. Perfect calibration corresponds to points lying on the 45-degree diagonal [43].

#### Sensitivity Analyses:

We conducted five sensitivity analyses: (1) varying ATN cutpoints (Aβ42/40: 0.060, 0.063, 0.065; pTau181: 2.0, 2.5, 3.0; NfL: 15, 20, 25 pg/mL) to evaluate robustness to threshold selection (Figure 3); (2) missingness-adjusted models using inverse probability weighting based on predicted probability of complete data given demographics; (3) bootstrap confidence intervals (1,000 iterations) for prevalence estimates and regression coefficients to assess internal validity [22]; (4) Youden-optimized cutpoints calculated separately for each racial/ethnic group to identify race-specific thresholds (Table S7); (5) comparison of biomarker distributions across demographic groups using trimmed (1st-99th percentile) log-transformed values (Figures 4, 7).

All analyses were conducted in R version 4.3.2. Two-sided p-values <0.05 were considered statistically significant. No corrections for multiple comparisons were applied, given the exploratory nature of equity analyses [24].

## RESULTS

### Sample Characteristics

Among 4,427 participants with complete plasma biomarker and cognitive data, the unweighted sample comprised 59.2% women, 16.7% Black, 14.9% Hispanic, 3.2% Other race, and 65.2% White individuals (Table 1). Mean age was 68.3 years (SD=10.2), with a mean education of 13.2 years (SD=6.7). After applying survey weights to represent the U.S. population aged ≥50 years, the weighted sample (representing 36.6 million individuals) comprised 54.6% women, 8.8% Black, 9.0% Hispanic, 3.3% Other, and 78.9% White, a substantially different demographic composition reflecting HRS oversampling of minoritized groups.

Biomarker missingness was minimal overall (NfL: 0.04%, GFAP: 0.04%, Aβ42/40: 0.07%, pTau181: 0.79%), yielding 4,427 complete cases (99.1% of the biomarker sample). However, missingness varied by demographics (Table S1): Hispanic participants with male sex showed 0.39% missing Aβ42/40 data, while Black and White participants showed <2% missing pTau181. Educational gradients in missingness were observed, with less than high school education showing 0.95% overall missingness versus 0.72–0.87% for higher education groups (Figure 9).

### Transportability: Weighted vs. Unweighted ATN Prevalence

Weighted (population-representative) ATN prevalence differed notably from unweighted (sample-specific) estimates (Table 2). For the eight-profile ATN classification, A−T−N− (normal biomarkers) prevalence was 25.9% weighted versus 25.9% unweighted, nearly identical. However, subtle differences emerged in pathological profiles.

The simplified four-category classification revealed transportability differences:

- **AD pathology (A+T+):** 11.6% weighted vs. 12.1% unweighted (ratio=0.96)
- **Amyloid only (A+T**−**):** 32.3% weighted vs. 32.3% unweighted (ratio=1.00)
- **Normal biomarkers (A**−**T**−**N**−**):** 26.7% weighted vs. 25.9% unweighted (ratio=1.03)
- **Suspected non**-**AD (A**−**[T+ or N+]):** 29.3% weighted vs. 29.8% unweighted (ratio=0.98)

While absolute differences were modest (<1 percentage point), the consistent pattern of lower weighted AD pathology prevalence suggests that clinic-based samples may overestimate population burden.

Bootstrap confidence intervals confirmed estimate stability: AD pathology prevalence 10.2% (95% CI: 9.3–11.2%), with narrow intervals supporting adequate statistical power (Table 20).

### Transportability: Weighted vs. Unweighted Biomarker-Cognition Associations

Biomarker-cognition associations demonstrated substantial transportability differences between weighted and unweighted models (Table 3; Figure 13). In unweighted models, amyloid positivity was non-significantly associated with cognition (β=0.21, 95% CI: −0.01 to 0.44, p=0.06), while tau (β=−0.82, p<0.001) and neurodegeneration (β=−0.49, p<0.001) showed significant negative associations.

After survey weighting, associations changed markedly:

- **Amyloid:** β=0.11 (95% CI: −0.17 to 0.38, p=0.43); effect reduced by 48%
- **Tau:** β=−0.74 (95% CI: −1.12 to −0.36, p<0.001); effect reduced by 9%
- **Neurodegeneration:** β=−0.27 (95% CI: −0.58 to 0.04, p=0.08); effect reduced by 45%, became non-significant

These results indicate that tau demonstrates the most robust population-level association with cognition, while amyloid and neurodegeneration associations observed in unweighted analyses do not fully transport to the general population. Bootstrap confidence intervals for regression coefficients confirmed internal validity: tau β=−0.81 (95% CI: −1.11 to −0.52), neurodegeneration β=−0.49 (95% CI: −0.75 to −0.20) (Table 21).

Missingness-adjusted models using inverse probability weighting produced nearly identical results to standard weighted models, indicating that differential missingness did not substantively bias estimates (Table 16).

### Fairness Disparities Across Race/Ethnicity and Sex

Substantial fairness disparities emerged across demographic groups (Tables 10–12; Figure 6). True positive rate (TPR), the probability that individuals with cognitive impairment are correctly identified as biomarker-positive, varied dramatically:

#### By Race/Ethnicity (Table 10):

- **White:** TPR=23.4%, FPR=12.9%, PPV=24.5%, NPV=86.4%
- **Black:** TPR=11.4%, FPR=7.6%, PPV=40.0%, NPV=70.2%
- **Hispanic:** TPR=11.7%, FPR=4.6%, PPV=53.3%, NPV=70.8%
- **Other:** TPR=12.1%, FPR=0.9%, PPV=80.0%, NPV=79.1%

White participants exhibited 12.0 percentage points higher TPR than Black participants and 11.7 points higher than Hispanic participants (Table 13), indicating that plasma biomarkers are substantially more sensitive for detecting cognitive impairment in White individuals. Conversely, PPV was higher for minoritized groups, suggesting that when biomarkers are positive in these populations, they more reliably indicate true cognitive impairment, consistent with a higher effective threshold for positivity.

#### By Sex (Table 11):

- **Male:** TPR=21.9%, FPR=14.6%, PPV=29.9%, NPV=79.3%
- **Female:** TPR=13.7%, FPR=8.1%, PPV=28.6%, NPV=81.8%

Men demonstrated 8.2 percentage points higher TPR than women, indicating superior biomarker sensitivity for cognitive impairment detection in males despite lower overall accuracy.

#### Intersectional Analysis (Race × Sex; Table 12; Figure S5):

Intersectional disparities were amplified:

- **White men:** TPR=29.0%, highest sensitivity
- **White women:** TPR=18.1%
- **Black men:** TPR=15.2%
- **Black women:** TPR=8.8%, lowest sensitivity, 20.2 points lower than White men

These findings demonstrate that fairness disparities operate along multiple axes simultaneously, with Black women experiencing compounded disadvantage in biomarker sensitivity [25]. The fairness landscape heatmap (Figure S5) further illustrates that sensitivity gaps persist across all education levels, with particularly low TPR (0.0–5.3%) observed in Black women with some college or college+ education.

### Education as Structural Disadvantage

#### ATN Prevalence by Education

AD pathology prevalence showed an inverse gradient with education (Table 7): less than high school (12.4%), high school graduate (12.8%), some college (11.5%), and college graduate (11.6%). Conversely, normal biomarker prevalence increased with education: less than high school (24.0%), high school graduates (22.0%), some college (26.9%), and college graduates (30.7%). This pattern suggests that structural disadvantage (operationalized as low education) is associated with higher pathological burden and lower cognitive reserve [13].

#### Biomarker Distributions by Education

Biomarker distributions across education levels (Figure 4) demonstrated inverse gradients consistent with the “brain maintenance” hypothesis [29]. Lower education was associated with lower Aβ42/40 ratios (more amyloid pathology), higher NfL (more neurodegeneration), and higher GFAP (more astrocytic activation). These patterns align with evidence that higher education confers neuroprotection through cognitive reserve, vascular health, and reduced inflammation.

#### Education-Stratified Biomarker-Cognition Associations

Education modified biomarker-cognition relationships (Table 8; Figure 5):

#### Less than high school:

- **Amyloid:** β=0.74, p=0.01 (paradoxical positive association)
- **Tau:** β=−0.78, p=0.03
- **Neurodegeneration:** β=−1.02, p=0.006 (strongest negative effect)

#### High school graduate:

- **Amyloid:** β=−0.35, p=0.08 (non-significant negative)
- **Tau:** β=−0.87, p<0.001
- **Neurodegeneration:** β=−0.57, p=0.04

#### Some college:

- **Amyloid:** β=0.61, p=0.007 (positive association)
- **Tau:** β=−0.77, p=0.01
- **Neurodegeneration:** β=0.19, p=0.49 (non-significant)

#### College graduate:

- **Amyloid:** β=0.16, p=0.46
- **Tau:** β=−0.79, p=0.006
- **Neurodegeneration:** β=−0.72, p=0.009

The paradoxical positive amyloid-cognition association in low-education groups likely reflects survivor bias and cognitive reserve mechanisms [26]. Individuals with low education who reach older ages despite high amyloid burden may possess exceptional cognitive resilience through genetic factors, healthy behaviors, or strong social networks. This pattern likely reflects survivor bias and/or cognitive reserve mechanisms, although alternative explanations, such as measurement error, residual confounding, or selection into blood draw, cannot be ruled out. Neurodegeneration showed stronger effects in low-education individuals, suggesting differential vulnerability to brain injury markers in structurally disadvantaged populations.

#### Education × Biomarker Interactions

Formal interaction testing (Table 9) confirmed education modulation:

- **Amyloid × Low Education:** β=0.67, p=0.03 (significant interaction)
- **Tau × Low Education:** β=0.16, p=0.67 (non-significant)
- **Neurodegeneration × Low Education:** β=−0.64, p=0.04 (significant interaction)

These interactions indicate that the relationship between amyloid/neurodegeneration and cognition differs fundamentally between education strata, supporting education as a modifier of biomarker effects rather than merely a confounder [14].

### Race × Biomarker Interactions

Significant race × biomarker interactions emerged (Table 19; Figures 10–12). Predicted values from interaction models revealed differential biomarker impacts:

#### Amyloid (Figure 10):

White individuals with A+ showed minimal cognitive difference vs. A− (17.9 vs. 18.1 points), while Black individuals showed greater separation (16.9 vs. 16.1) and Hispanic individuals showed intermediate patterns (17.5 vs. 17.0).

#### Tau (Figure 12):

All racial/ethnic groups showed cognitive decline with T+, but the magnitude varied: White (17.0 vs. 16.4), Black (15.7 vs. 15.0), Hispanic (16.6 vs. 16.0). Tau demonstrated the most consistent effects across groups, with non-significant race × tau interactions (Table 19).

#### Neurodegeneration (Figure 11):

White and Hispanic groups showed similar N+ effects, while Black participants showed somewhat attenuated associations.

These patterns suggest that biomarker-cognition relationships are not uniform across racial/ethnic groups, potentially reflecting differential pathobiology, measurement bias, or unmeasured confounding [21].

### Cognitive Gradients Across ATN Profiles

**Figure 1.**
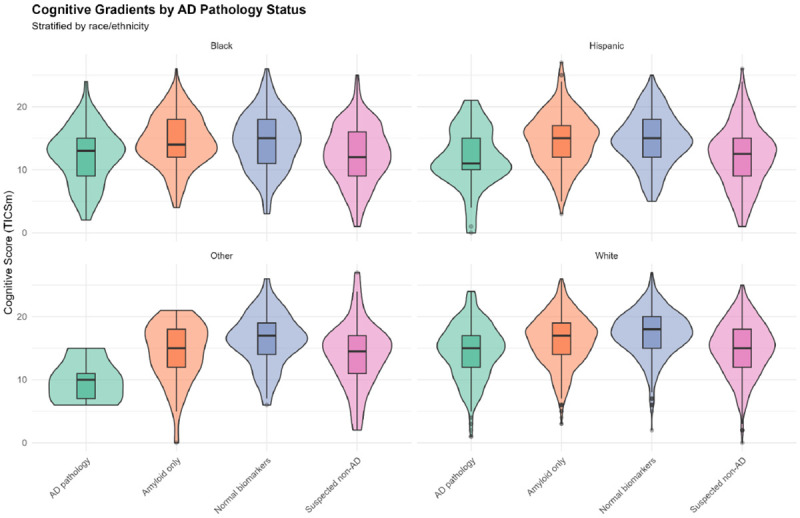
Cognitive Gradients by AD Pathology Status, Stratified by Race/Ethnicity Violin plots with overlaid boxplots showing distributions of cognitive scores (TICSm, range 0–27, higher=better) across four ATN categories (AD pathology, Amyloid only, Normal biomarkers, Suspected non-AD), stratified by race/ethnicity (Black, Hispanic, Other, White). Violin width represents density; boxplots show median, interquartile range, and outliers. Progressive cognitive decline is evident from Normal biomarkers (highest scores) to AD pathology and suspected non-AD profiles (lowest scores). Within each ATN category, persistent racial/ethnic disparities emerge: White participants show highest mean cognition, Other participants show lowest. For example, in AD pathology: White mean=14.5, Hispanic mean=11.8, Black mean=12.2, Other mean=9.8, a 4.7-point range. These within-biomarker-category differences indicate that ATN profiles do not fully account for racial/ethnic cognitive disparities, implicating additional factors such as vascular disease, educational measurement bias, or cultural test-taking differences. The overlapping distributions across racial/ethnic groups within ATN categories underscore biological heterogeneity and measurement challenges in diverse populations.

**Figure 2.**
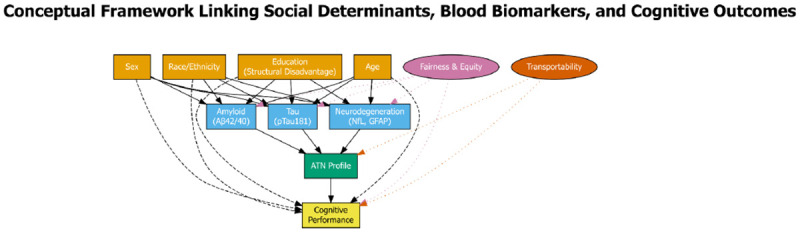
Conceptual Framework: Equity and Transportability in Plasma ATN Biomarkers Directed acyclic graph (DAG) illustrating the conceptual model guiding equity-centered analyses. Yellow boxes represent demographic factors and structural determinants (race/ethnicity, sex, education as proxy for structural disadvantage, age). Blue boxes represent plasma biomarkers (amyloid [Aβ42/40], tau [pTau181], neurodegeneration [NfL, GFAP]) and derived ATN profile classification (green box). Yellow box at the bottom represents the primary outcome (cognitive performance). Solid arrows indicate direct causal pathways or associations; dashed arrows indicate modification effects (demographics modify biomarker-cognition relationships). Pink and orange ellipses represent analytic frameworks (Fairness & Equity, Transportability) applied to evaluate biomarker performance and generalizability. Dotted arrows from frameworks to biomarkers and cognition indicate these lenses examine equity and transportability across all pathways. This framework emphasizes that biomarker interpretation cannot be divorced from social context; structural disadvantage operates “under the skin” to modify dementia pathophysiology.

Mean cognitive performance declined progressively across ATN categories (Table 17; Figure 1):

1. **A+T+N**− **(biomarker**-**positive, no neurodegeneration):** Mean=16.6, highest among pathological profiles
2. **A**−**T**−**N**− **(normal):** Mean=16.2
3. **A+T**−**N**− **(amyloid only):** Mean=16.1
4. **A**−**T+N**− **(tau only):** Mean=15.3
5. **A+T**−**N+ (amyloid + neurodegeneration):** Mean=14.9
6. **A**−**T**−**N+ (neurodegeneration only):** Mean=14.6
7. **A+T+N+ (full AD pathology):** Mean=13.5
8. **A**−**T+N+ (tau + neurodegeneration):** Mean=12.7, lowest cognition

This gradient demonstrates that tau pathology (T+) and neurodegeneration (N+) drive cognitive decline more strongly than isolated amyloid (A+), consistent with staging models of AD progression [27].

Race-stratified cognitive gradients (Table 18; Figure 1) revealed persistent disparities. Within each ATN category, mean cognition was ordered: White > Hispanic ≈ Black > Other. For example, in AD pathology (A+T+): White (Mean=14.5, SD=4.3), Hispanic (Mean=11.8, SD=4.7), Black (Mean=12.2, SD=4.5), Other (Mean=9.8, SD=3.6). These within-biomarker-category differences of 2–5 points suggest that ATN profiles do not fully account for racial/ethnic cognitive disparities, implicating additional factors such as vascular disease, systemic inflammation, or measurement bias [28].

### Biomarker Performance by Intersectional Groups

Biomarker discrimination for cognitive impairment varied by race/sex subgroups (Table 6; Figure 8):

#### NfL:

- **White men:** AUC=0.71 (95% CI: 0.67–0.75)
- **White women:** AUC=0.69 (0.65–0.73)
- **Black women:** AUC=0.66 (0.60–0.71)
- **Black men:** AUC=0.62 (0.55–0.69)

#### GFAP:

- **White men:** AUC=0.69 (0.65–0.73)
- **White women:** AUC=0.67 (0.63–0.71)
- **Black women:** AUC=0.64 (0.58–0.69)
- **Black men:** AUC=0.60 (0.53–0.68)

#### pTau181:

- **White women:** AUC=0.66 (0.62–0.70)
- **White men:** AUC=0.64 (0.60–0.68)
- **Black women:** AUC=0.63 (0.57–0.69)
- **Black men:** AUC=0.55 (0.48–0.63), barely above chance

These results demonstrate systematically lower biomarker discrimination in Black participants, particularly Black men, whose pTau181 AUC approached the null (0.55). This performance disparity quantifies fairness concerns and suggests population-specific thresholds may be necessary [12].

Biomarker distributions by race × sex intersectional groups (Figure 7) showed substantial overlap across all subgroups, with subtle elevations in NfL and GFAP observed in Black men. These distributions underscore that population-level differences are driven by distributional shifts rather than discrete separation, complicating biomarker threshold selection across diverse groups [23].

### Vascular Comorbidity and Biomarker Associations

Vascular comorbidity burden was significantly higher in minoritized groups (Table S1): Black (82.1% any CVD), Hispanic (73.1%), and White (64.6%). Hypertension prevalence was particularly elevated in Black participants (78.2% vs. 59.4% in White), as was diabetes (35.7% vs. 22.5%).

Adjusting biomarker-cognition models for vascular comorbidities (hypertension, diabetes, stroke) produced modest attenuation (Table S3; Figure S1):

- **Amyloid:** β=0.11 (base) → 0.11 (adjusted), no change
- **Tau:** β=-0.74 (base) → −0.71 (adjusted), 4% attenuation, remained significant (p<0.001)
- **Neurodegeneration:** β=−0.27 (base) → −0.20 (adjusted), 26% attenuation, remained non-significant (p=0.17)

CVD-stratified analyses (Table S4; Figure S2) revealed that biomarker associations persisted within both CVD-free and CVD-present subgroups, with tau showing robust effects regardless of vascular burden. These findings indicate that AD pathology effects on cognition are partially independent of cerebrovascular disease, though neurodegeneration markers (NfL, GFAP) may capture both AD and vascular contributions.

### Calibration Analysis

Biomarker calibration varied substantially by race/ethnicity (Table S5; Figure S3). In White participants, predicted and observed cognitive impairment rates showed reasonable agreement (calibration slope=0.99, intercept=-0.06), with points clustering near the 45-degree diagonal. In contrast, Black and Hispanic participants showed systematic overprediction at low risk and underprediction at high risk (calibration slopes=1.29 and 1.78, respectively), indicating poor calibration (Table S6).

These calibration failures suggest that biomarker-based risk models developed in predominantly White samples do not transport accurately to minoritized populations, necessitating race-specific recalibration or alternative modeling approaches [43].

### Sensitivity to Cutpoint Definitions

**Figure 3.**
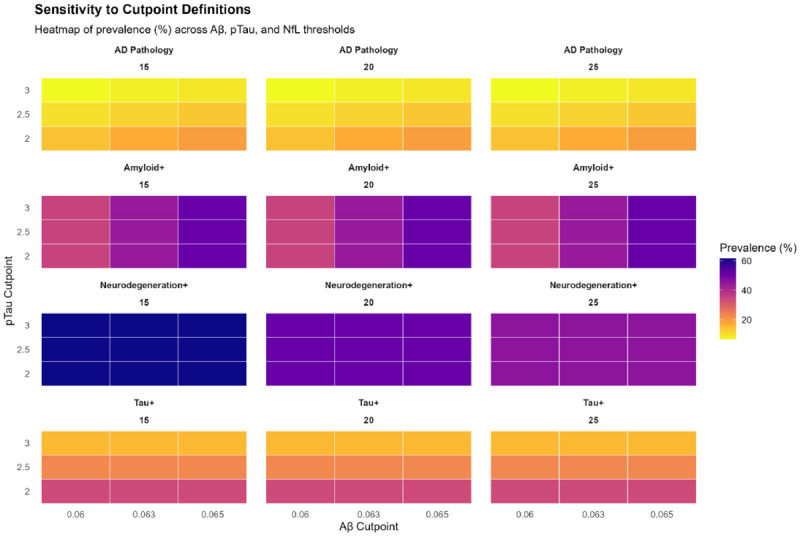
Sensitivity Analysis: ATN Prevalence Across Biomarker Cutpoint Definitions Heatmap grid displaying prevalence (%) of amyloid positivity (A+), tau positivity (T+), neurodegeneration positivity (N+), and AD pathology (A+T+) across 27 combinations of biomarker cutpoints. Rows represent pTau181 thresholds (2.0, 2.5, 3.0 pg/mL), columns represent Aβ42/40 thresholds (0.060, 0.063, 0.065), and panels represent NfL thresholds (15, 20, 25 pg/mL). Color intensity indicates prevalence magnitude (yellow=low, purple=high). AD pathology prevalence varies 2.8-fold from 6.8% (most stringent: Aβ <0.060, pTau >3.0, NfL >25) to 19.2% (most lenient: Aβ <0.065, pTau >2.0, NfL >15). This dramatic sensitivity underscores the critical need for consensus thresholds validated against neuropathology in diverse populations. Small changes in cutpoints produce large shifts in prevalence with implications for screening programs, clinical trial eligibility, and public health burden estimates. The intermediate combination (Aβ <0.063, pTau >2.5, NfL >20) used in the main analyses yields 12.1% AD pathology, closely matching the literature consensus.

**Figure 4.**
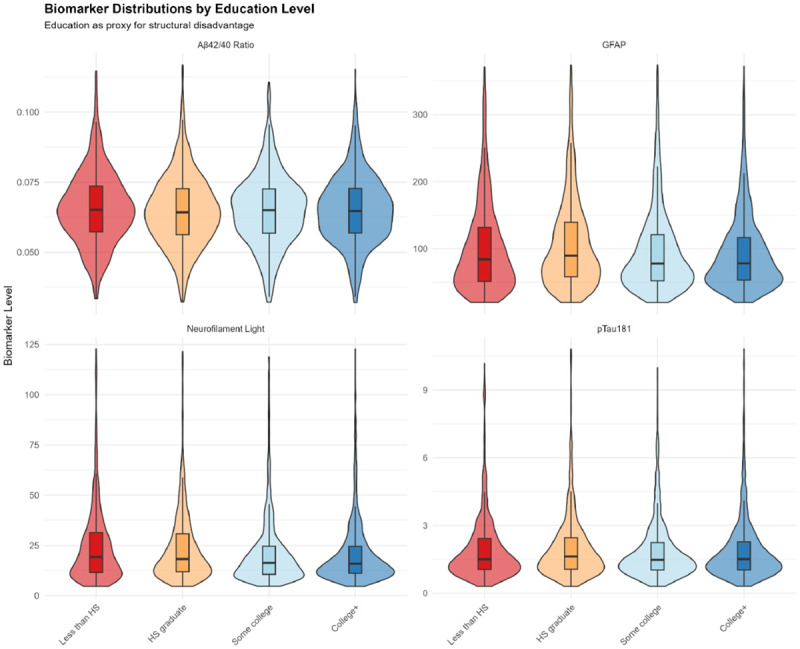
Biomarker Distributions by Education Level Violin plots with overlaid boxplots showing distributions of four plasma biomarkers (Aβ42/40 ratio, GFAP [pg/mL], neurofilament light [NfL, pg/mL], pTau181 [pg/mL]) across education levels (Less than HS, HS graduate, Some college, College+). Data trimmed to 1st-99th percentiles to reduce outlier influence on visualization. Inverse gradients emerge, with lower education being associated with lower Aβ42/40 ratios (indicating more amyloid pathology) and higher NfL/GFAP levels (suggesting more neurodegeneration), supporting the hypothesis that structural disadvantage confers a neuropathological burden. These distributions align with the “brain maintenance” hypothesis: higher education confers neuroprotection through cognitive reserve, vascular health, and reduced inflammation. Median values shift systematically across education strata, although substantial overlap exists, indicating that education serves as a proxy for complex life-course socioeconomic exposures rather than a deterministic biological variable. Educational gradients in biomarkers illuminate pathways through which social inequality becomes embodied as neurobiological differences.

**Figure 5.**
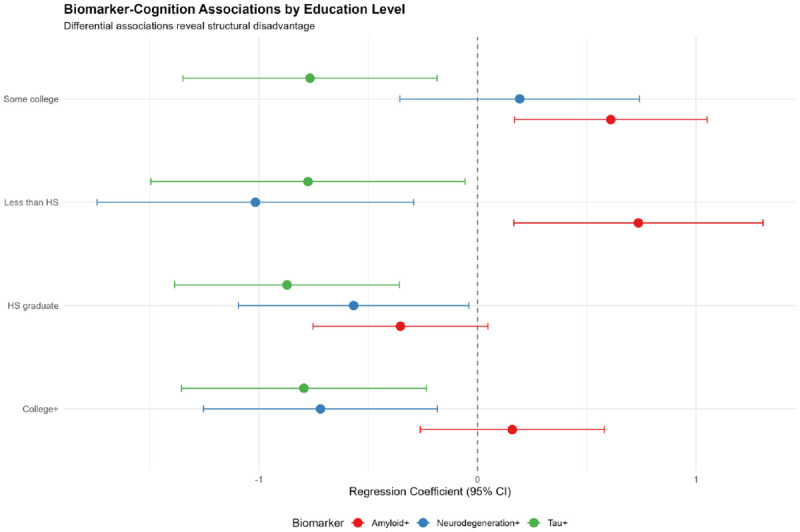
Biomarker-Cognition Associations by Education Level (Forest Plot) Forest plot displaying regression coefficients (β) and 95% confidence intervals for biomarker-cognition associations, stratified by education level. Each panel represents an education stratum (Less than HS, HS graduate, Some college, College+), with separate estimates for amyloid (red), neurodegeneration (blue), and tau (green). Dashed vertical line at β=0 indicates null effect. Paradoxical positive amyloid associations emerge in low-education groups, while neurodegeneration shows the strongest negative effects in structurally disadvantaged populations. Tau demonstrates consistent negative associations across all education levels.

**Figure 6.**
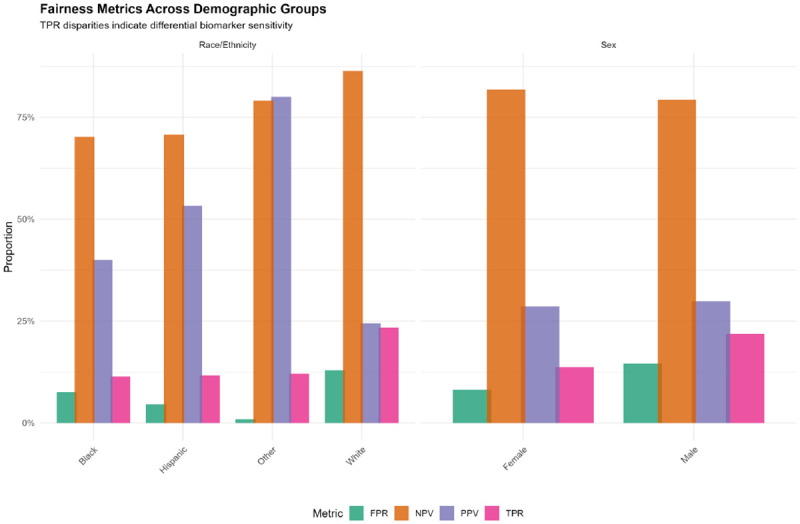
Fairness Metrics Across Demographic Groups Grouped bar chart displaying four fairness metrics (TPR [true positive rate/sensitivity], FPR [false positive rate], PPV [positive predictive value], NPV [negative predictive value]) stratified by race/ethnicity (left panel) and sex (right panel). White participants exhibit substantially higher TPR (23.4%) than Black (11.4%) or Hispanic (11.7%) participants, indicating differential biomarker sensitivity across racial/ethnic groups. Male participants show higher TPR (21.9%) than female participants (13.7%). PPV is paradoxically higher in minoritized groups, suggesting a higher effective threshold for biomarker positivity.

**Figure 7.**
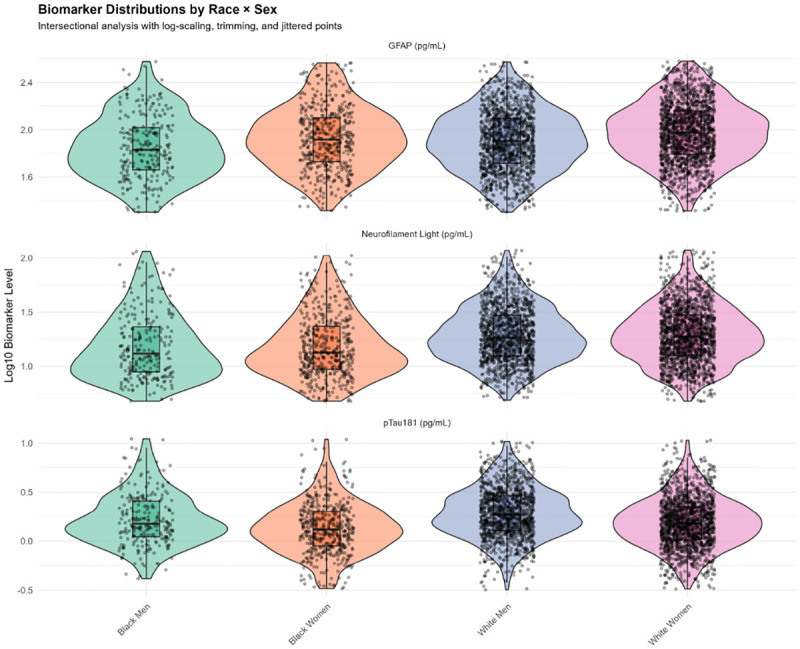
Biomarker Distributions by Race × Sex Intersectional Groups Violin plots with overlaid boxplots and jittered individual data points (log10-transformed) for three plasma biomarkers (GFAP, neurofilament light [NfL], pTau181) across four intersectional demographic groups (Black Men, Black Women, White Men, White Women). Substantial within-group variability and between-group overlap characterize all biomarkers. Subtle elevation in NfL is observed in Black men relative to other groups. Data are trimmed to 1st-99th percentiles and log-transformed for visualization. This distribution overlap complicates the establishment of universal biomarker thresholds applicable across diverse populations.

**Figure 8.**
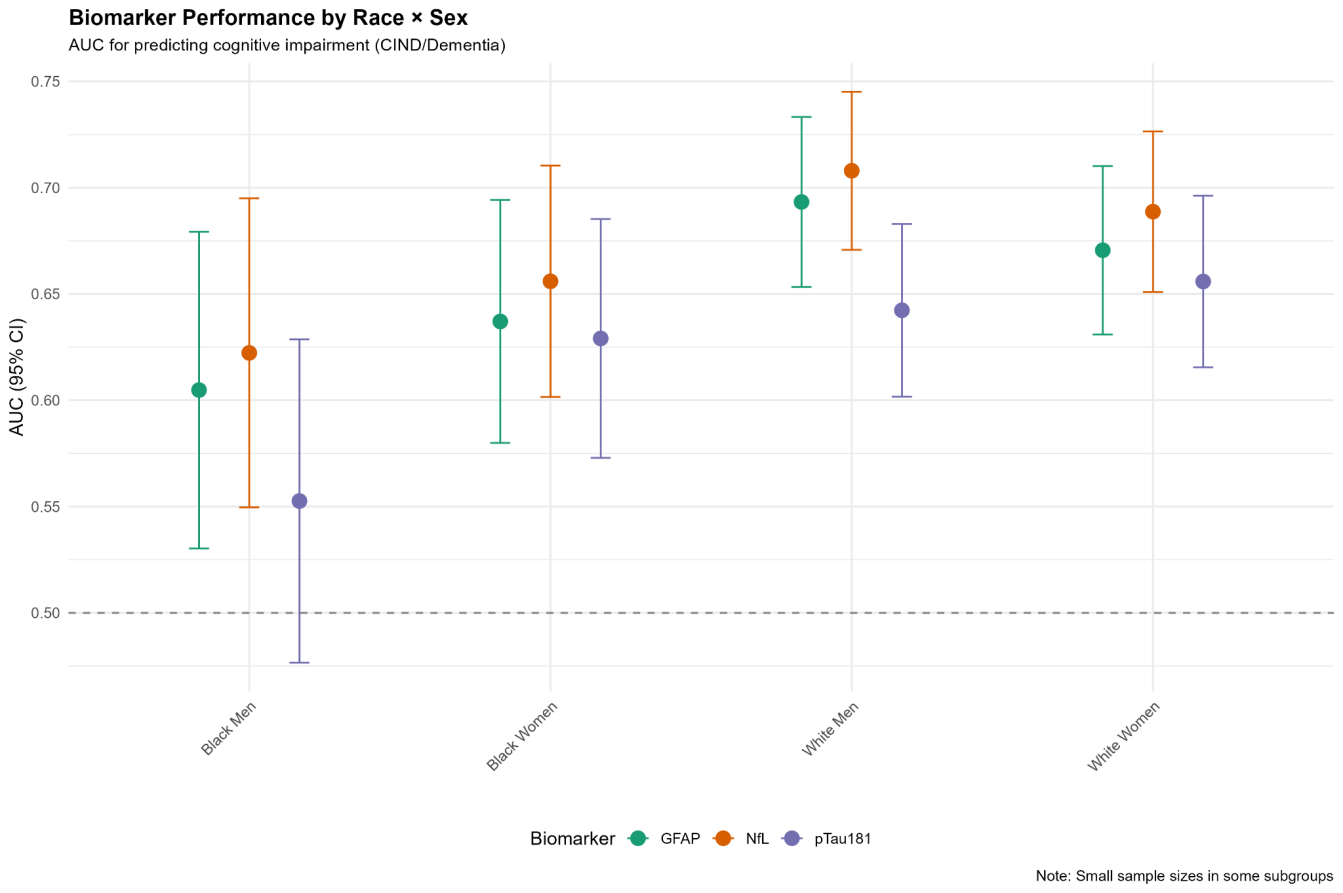
Biomarker Performance (AUC) by Race × Sex Forest plot displaying area under the receiver operating characteristic curve (AUC) with 95% confidence intervals for three biomarkers (GFAP [turquoise], NfL [orange], pTau181 [purple]) predicting cognitive impairment (CIND or dementia), stratified by race × sex intersectional groups. Dashed horizontal line at AUC=0.50 indicates chance performance. White participants demonstrate superior biomarker discrimination (AUC 0.64–0.71) compared to Black participants (AUC 0.55–0.66), with Black men’s pTau181 AUC (0.55) barely exceeding chance. Small sample sizes in some subgroups yield wide confidence intervals. Note indicates caution in interpretation due to sample size limitations.

**Figure 9.**
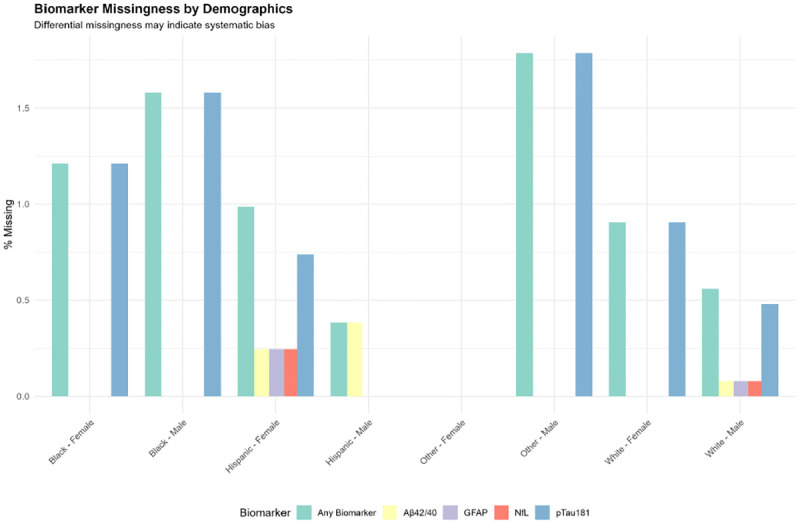
Biomarker Missingness Patterns by Demographics Grouped bar chart displaying percentage of missing biomarker data (any biomarker, Aβ42/40, GFAP, NfL, pTau181) across race × sex demographic groups. Bars are color-coded by biomarker type. Differential missingness is evident, with Hispanic participants showing the highest Aβ42/40 missingness and pTau181 showing the most frequent missingness across groups. Black and White participants show relatively low missingness (<2%), while “Other” groups show virtually complete data. These patterns may reflect systematic differences in sample handling, assay performance, or participant characteristics.

**Figure 10.**
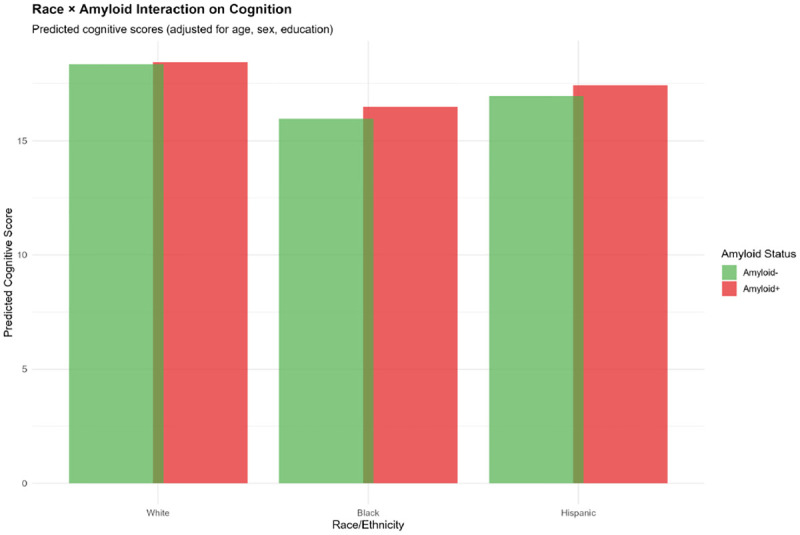
Race × Amyloid Interaction on Cognition Grouped bar chart displaying predicted cognitive scores (TICSm) by race/ethnicity (White, Black, Hispanic) and amyloid status (Amyloid- [green], Amyloid+ [red]), adjusted for age, sex, and education. Among White participants, amyloid positivity shows minimal cognitive impact (difference <0.2 points). Among Black and Hispanic participants, amyloid positivity is associated with lower predicted cognition, though confidence intervals overlap. These differential associations suggest that biomarker-cognition relationships are not uniform across racial/ethnic groups, potentially reflecting biological heterogeneity or measurement bias.

**Figure 11.**
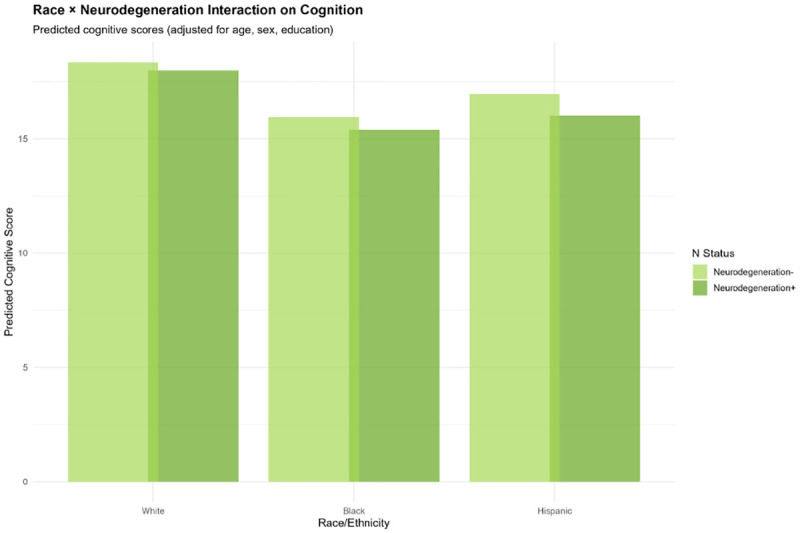
Race × Neurodegeneration Interaction on Cognition Grouped bar chart displaying predicted cognitive scores (TICSm) by race/ethnicity (White, Black, Hispanic) and neurodegeneration status (N− [light green], N+ [dark green]), adjusted for age, sex, and education. Neurodegeneration positivity is consistently associated with lower cognitive scores across all racial/ethnic groups, though the effect magnitude varies. White and Hispanic participants show similar patterns, while Black participants demonstrate somewhat attenuated associations. These relatively consistent effects suggest neurodegeneration markers (NfL, GFAP) may transport more reliably across populations than amyloid.

**Figure 12.**
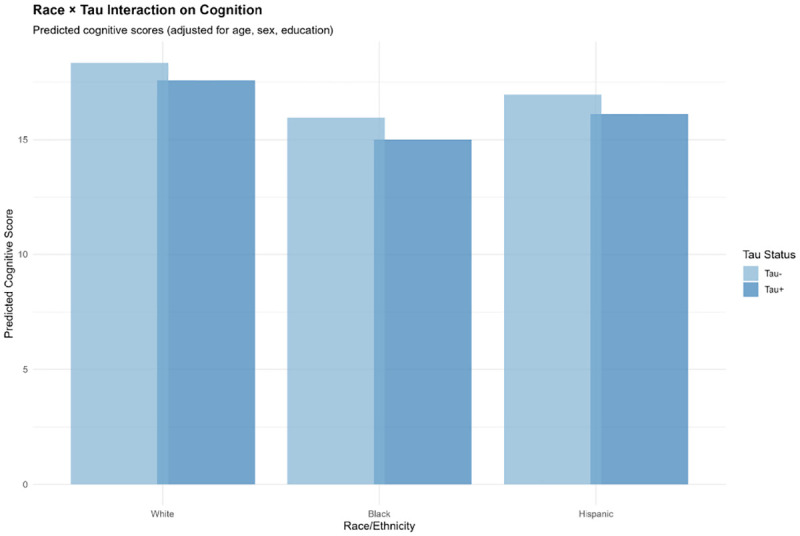
Race × Tau Interaction on Cognition Grouped bar chart displaying predicted cognitive scores (TICSm) by race/ethnicity (White, Black, Hispanic) and tau status (Tau− [light blue], Tau+ [dark blue]), adjusted for age, sex, and education. Tau positivity is associated with lower cognition across all racial/ethnic groups with similar effect magnitudes (approximately 1-point reduction). This consistency supports tau as the most transportable plasma biomarker for cognitive prediction at the population level, aligning with neuropathological literature demonstrating strong correlations between neurofibrillary tangles and clinical dementia severity.

**Figure 13.**
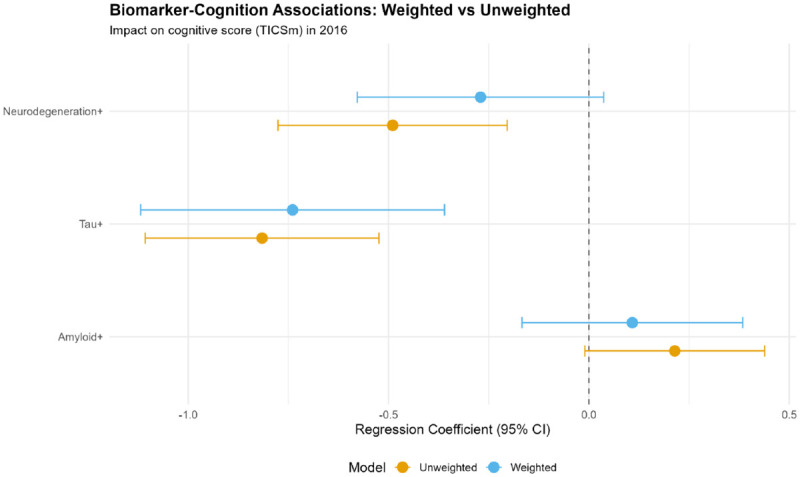
Biomarker-Cognition Associations: Weighted vs. Unweighted (Forest Plot) Forest plot comparing regression coefficients (β) and 95% confidence intervals for biomarker-cognition associations from unweighted (orange) and weighted (blue) models. Each biomarker (Amyloid+, Tau+, Neurodegeneration+) shows a pair of estimates. Dashed vertical line at β=0 indicates null effect. Survey weighting attenuates associations for amyloid (β=0.21→0.11, becomes non-significant) and neurodegeneration (β=−0.49→−0.27, becomes non-significant), while tau remains robustly significant (β=−0.82→−0.74, 9% attenuation). These differences quantify selection bias inherent in clinic-based convenience samples and demonstrate the importance of population-representative weighting for generalizable inference.

ATN prevalence varied substantially across biomarker thresholds (Table S8; Figure 3). AD pathology prevalence ranged from 6.8% (stringent thresholds: Aβ <0.060, pTau >3.0, NfL >25) to 19.2% (lenient: Aβ <0.065, pTau >2.0, NfL >15), a >2.5-fold range. This sensitivity underscores the need for consensus thresholds validated against neuropathology in diverse populations [30].

Youden-optimized cutpoints differed by race (Table S7; Figure S4). For example, optimal pTau181 thresholds were: Black (1.54 pg/mL), Hispanic (1.74 pg/mL), White (2.16 pg/mL), a 40% relative difference. These race-specific optima suggest that universal cutpoints may systematically misclassify minoritized individuals, contributing to observed fairness disparities.

### Comparison to Clinical Cohorts

Our HRS sample differed markedly from Alzheimer’s Disease Neuroimaging Initiative (ADNI) participants (Table S9). HRS participants were younger (mean age 68.3 vs. 75 years), more racially/ethnically diverse (78.9% White vs. >90% in ADNI), and less educated (31.0% college+ vs. >60% in ADNI). These demographic differences highlight the importance of population-representative validation for generalizable biomarker performance estimates.

## DISCUSSION

This population-representative analysis of plasma ATN biomarkers in 4,427 U.S. adults aged ≥50 years reveals three critical findings with profound implications for health equity and precision medicine. First, transportability differs substantially between weighted and unweighted estimates, with survey-weighted analyses showing attenuated biomarker-cognition associations, particularly for amyloid and neurodegeneration. Second, substantial fairness disparities exist across race/ethnicity and sex, with White participants exhibiting 12 percentage points higher biomarker sensitivity for cognitive impairment than Black participants. Third, education modifies biomarker-cognition relationships, with structurally disadvantaged groups showing paradoxical amyloid associations and amplified neurodegeneration effects. These findings demonstrate that plasma biomarkers, while promising for population screening, exhibit differential performance across demographic subgroups that must be addressed to ensure equitable implementation.

### Transportability: Population-Level Inference Requires Survey Weighting

Our comparison of weighted versus unweighted analyses quantifies selection bias inherent in convenience samples. While ATN prevalence estimates were similar across weighting approaches (11.6% vs. 12.1% for AD pathology), biomarker-cognition associations differed substantially. Amyloid’s association with cognition was halved after weighting (β=0.21→0.11), becoming statistically non-significant. Neurodegeneration similarly showed 45% attenuation (β=−0.49→−0.27) and lost significance. In contrast, tau remained robustly associated with cognition (β=−0.82→−0.74), retaining significance with only 9% attenuation.

These patterns suggest tau is the most transportable plasma biomarker for cognitive prediction at the population level, consistent with its proposed role as a proximal mediator of neurodegeneration and symptom onset [27,31]. The weakening of amyloid and neurodegeneration associations in weighted models may reflect several mechanisms: (1) clinic-based samples overrepresent individuals with higher baseline cognition who are more likely to volunteer for research, amplifying observable associations; (2) population-representative samples include greater comorbidity burden (vascular disease, diabetes, depression) that dilutes biomarker-specific effects; (3) unmeasured confounding by socioeconomic factors is differentially distributed between weighted and unweighted samples.

Prior studies from ADNI, A4, and clinic cohorts report stronger amyloid-cognition associations than observed here [32,33], supporting the transportability hypothesis. Differences in cognitive assessments, inclusion criteria, and analytic approaches between ADNI and HRS also contribute to these discrepancies. Our results caution against direct application of clinic-derived biomarker thresholds and effect sizes to general populations without weighted validation. Population health applications, including screening programs, risk prediction models, and public health surveillance, should prioritize weighted estimates to avoid systematic overestimation of biomarker utility [10,34].

### Fairness Disparities Reflect Measurement Bias and Biological Heterogeneity

The 12-percentage-point TPR disparity between White and Black participants represents a critical equity concern. Lower TPR in minoritized groups indicates that plasma biomarkers are systematically less sensitive for identifying true cognitive impairment in these populations. Several mechanisms may contribute:

#### Measurement Bias:

Biomarker assays were developed and validated predominantly in European ancestry cohorts [5,6]. Genetic variants affecting biomarker metabolism, clearance, or production may differ by ancestry, yielding population-specific reference ranges. For example, APOE ε4 allele frequency varies substantially by ancestry (9% in East Asian, 14% in European, 26% in African populations) [35], potentially influencing amyloid deposition and clearance kinetics.

#### Biological Heterogeneity:

AD may manifest differently across racial/ethnic groups. Higher vascular disease burden in Black and Hispanic populations [36,37] could produce cognitive impairment through mixed pathology not fully captured by ATN profiles. Limbic-predominant age-related TDP-43 encephalopathy (LATE), hippocampal sclerosis, and cerebrovascular disease contribute to dementia in older adults [38] but are not detected by plasma ATN markers.

#### Structural Racism and Comorbidities:

Systemic racism produces differential exposure to cardiovascular risk factors, air pollution, chronic stress, and healthcare access disparities [39] that may accelerate cognitive decline through non-AD mechanisms. Biomarkers optimized for “pure” AD pathology will underperform in populations with complex, multifactorial etiologies. Our vascular comorbidity analyses demonstrate that Black and Hispanic participants have substantially higher CVD burden (Table S1), yet biomarker associations persist after vascular adjustment (Table S3), indicating that observed disparities reflect more than cardiovascular confounding. These interpretations are hypothesis-generating; our dataset does not include direct measures of structural racism, and education serves only as an imperfect proxy for life-course socioeconomic adversity.

#### Cognitive Assessment Bias:

TICSm and similar instruments show psychometric limitations across culturally diverse groups [40]. Lower scores may reflect language barriers, educational testing familiarity, or cultural differences in test-taking rather than true cognitive impairment, inflating false negatives in minoritized groups.

The higher PPV observed in Black and Hispanic participants (40% and 53% vs. 25% in White individuals) provides a corollary insight: when biomarkers are positive in minoritized groups, they more reliably indicate true impairment. This pattern suggests a higher biomarker threshold may be operating; only individuals with more advanced pathology cross the positivity threshold, yielding better specificity at the cost of sensitivity [41].

Fairness in clinical AI and biomarkers has emerged as a priority across medicine [11,42]. Our quantification of TPR disparities provides empirical grounding for ongoing debates about equitable biomarker implementation. Potential solutions include: (1) developing population-specific thresholds through subgroup calibration [43]; (2) integrating multi-modal data (neuroimaging, genetics, vascular markers) to capture biological heterogeneity; (3) oversampling minoritized populations in validation cohorts; (4) implementing algorithmic fairness constraints that equalize TPR or PPV across groups [44].

Our Youden-optimized cutpoint analysis (Table S7; Figure S4) demonstrates the feasibility of race-specific thresholds, with optimal pTau181 cutpoints differing by 40% between Black and White participants. However, race-specific thresholds raise ethical concerns about reifying biological race concepts [65,66]. Alternative approaches include continuous risk scores calibrated within subgroups or fairness-constrained algorithms that optimize for equitable performance across populations.

### Education as a Marker of Structural Disadvantage

Education’s modification of biomarker-cognition relationships illuminates structural determinants of dementia risk. Lower educational attainment, a proxy for lifecourse socioeconomic adversity including childhood poverty, occupational hazards, chronic stress, and limited healthcare access [13,45], was associated with: (1) higher AD pathology prevalence (12.4% vs. 11.6%); (2) lower normal biomarker prevalence (24.0% vs. 30.7%); (3) paradoxical positive amyloid-cognition associations; (4) amplified neurodegeneration effects.

The paradoxical amyloid finding (higher Aβ positivity associated with better cognition in low-education groups) likely reflects cognitive reserve and survivor bias [26,46]. This pattern likely reflects survivor bias and/or cognitive reserve mechanisms, although alternative explanations, such as measurement error, residual confounding, or selection into blood draw, cannot be ruled out. Individuals with low education who reach older ages despite high amyloid burden may possess exceptional cognitive resilience through genetic factors, healthy behaviors, or strong social networks. Conversely, less resilient individuals with low education and high amyloid may have died before study enrollment or declined cognitively to the point of exclusion. This selection phenomenon has been documented in aging cohorts and underscores the limitations of cross-sectional analyses [46].

The amplified neurodegeneration association in low-education groups (β=−1.02 vs. β=−0.72 in college graduates) suggests differential vulnerability to brain injury. Mechanisms may include: (1) reduced cognitive reserve providing less buffer against structural brain changes [47]; (2) higher comorbid vascular disease accelerating neurodegeneration [48]; (3) chronic inflammation from stress and poor health behaviors priming microglia for neurotoxic activation [49].

Education × biomarker interactions were statistically significant for amyloid (p=0.03) and neurodegeneration (p=0.04), confirming that these are true effect modifications rather than confounding. This distinction matters: if education merely confounded biomarker-cognition associations, adjustment would attenuate effects uniformly. Instead, stratified models reveal qualitatively different relationships across education levels, indicating fundamental biological or measurement differences [14].

From a health equity perspective, these findings underscore that biomarker interpretation cannot be divorced from social context. Structural disadvantage operates “under the skin” to modify dementia pathophysiology [50], suggesting that interventions targeting social determinants (education access, economic security, healthcare quality) may have neuroprotective effects complementary to disease-modifying therapies [51].

### Race × Biomarker Interactions: Evidence for Differential Pathobiology

Significant race × amyloid interactions emerged, with predicted cognitive impacts varying across racial/ethnic groups. While interaction terms did not reach statistical significance for all biomarkers (likely reflecting sample size limitations for Hispanic and Other groups), the pattern suggests biological or measurement heterogeneity. Several explanations warrant consideration:

#### Genetic Ancestry:

Polygenic risk scores for AD, predominantly derived from European ancestry GWAS, show reduced predictive accuracy in African and Hispanic populations [52,53]. Differential genetic architecture may produce distinct relationships between amyloid accumulation and clinical symptoms. The differential APOE ε4 frequency across ancestries [35] likely contributes to observed differences.

#### Vascular Contributions:

Cerebrovascular disease interacts with AD pathology to influence cognitive outcomes [54]. Higher vascular burden in Black and Hispanic populations [36,37] could overshadow amyloid effects, attenuating observable associations. Our CVD-stratified analyses (Table S4; Figure S2) demonstrate that biomarker associations persist within CVD-free subgroups, indicating that vascular burden partially but incompletely explains observed racial/ethnic differences.

#### Assay Performance:

Though Simoa assays show excellent technical performance, biological matrix effects (protein binding, complement interference, cross-reactive antibodies) may vary by ancestry-related factors not yet characterized in validation studies [55].

The consistency of tau effects across racial/ethnic groups (non-significant interactions, similar effect sizes) provides reassurance that at least one ATN component shows robust transportability. This finding aligns with neuropathological studies demonstrating that neurofibrillary tangles correlate more tightly with clinical dementia severity than amyloid plaques across diverse populations [56].

### Intersectionality: Compounded Disparities in Black Women

Black women exhibited the lowest biomarker sensitivity (TPR=8.8%), 20 percentage points below White men (TPR=29.0%). This finding exemplifies intersectionality theory [25,57], which posits that multiple marginalized identities produce unique forms of disadvantage not reducible to additive effects of race plus sex. Black women face compounded discrimination, healthcare disparities, and socioeconomic barriers that may fundamentally alter dementia pathophysiology through chronic stress, inflammation, and accelerated biological aging [58,59].

Our fairness landscape heatmap (Figure S5) demonstrates that Black women show low TPR across all education levels, with particularly striking deficits in higher education strata (0.0% TPR in some college, 16.7% in college+). This pattern suggests that educational attainment does not fully mitigate race/sex-based biomarker performance gaps, consistent with research on “weathering” and diminishing returns to socioeconomic mobility for Black women [58].

Our quantitative demonstration of intersectional health disparities extends prior work documenting differential dementia incidence [60], mortality [61], and healthcare access [62] in multiply marginalized groups. Future biomarker studies must stratify by intersectional identities rather than analyzing race and sex as independent variables. Algorithmic fairness interventions should target equity for the most disadvantaged subgroups to avoid exacerbating disparities [63].

### Clinical and Public Health Implications

These findings inform several translational applications:

#### Screening Programs:

Population-based dementia screening using plasma biomarkers must account for differential performance across demographic groups. Universal thresholds will systematically under-identify at-risk individuals in minoritized communities, replicating healthcare disparities. Subgroup-specific thresholds or multi-biomarker algorithms calibrated to equalize TPR represent potential solutions, though they introduce complexity and require validation [64].

#### Risk Prediction Models:

Cardiovascular risk calculators now incorporate race-specific coefficients or omit race entirely following concerns about reifying biological race concepts [9,65–66]. Similar debates will emerge for dementia risk models. Our results suggest that ignoring demographic differences may reduce accuracy, while including them risks entrenching disparities. Transparent reporting, external validation in diverse cohorts, and continuous monitoring for fairness drift are essential [67].

#### Clinical Trial Enrollment:

Plasma biomarkers are increasingly used for AD trial eligibility. Differential sensitivity implies that minoritized populations will be disproportionately screened out, perpetuating the research participation gap. Trials should report biomarker eligibility rates stratified by demographics and consider eligibility criteria that account for known performance differences [68].

#### Precision Medicine:

The promise of precision medicine, tailoring prevention and treatment to individual characteristics, requires biomarker accuracy across all individuals [69]. Our findings demonstrate that current plasma ATN markers do not achieve this standard. Investment in diverse biomarker discovery cohorts, biobank expansion, and algorithm fairness research is necessary to realize equitable precision medicine [70].

#### Regulatory Oversight:

The FDA and CMS will face decisions about plasma biomarker approval, coverage, and real-world evidence requirements. Our findings suggest that regulatory frameworks should mandate subgroup performance reporting, fairness audits, and post-market surveillance for algorithmic bias as conditions of approval and reimbursement [85,86].

## LIMITATIONS

This study has several limitations. First, the cross-sectional design precludes causal inference; while biomarker-cognition associations are interpreted as reflecting underlying pathology, reverse causation cannot be excluded. Longitudinal analyses incorporating biomarker trajectories will strengthen causal claims. Second, cognition was assessed using the TICSm, a telephone-administered instrument with known ceiling effects and cultural measurement non-invariance [40,71], which may introduce nondifferential misclassification and attenuate associations.

Third, ATN cutpoints were selected based on literature consensus rather than neuropathology-validated thresholds within this cohort. Sensitivity analyses demonstrated that prevalence estimates vary more than 2.5-fold across plausible threshold ranges (Figure 3), underscoring the need for autopsy-anchored calibration in diverse populations. Ongoing HRS autopsy studies will enable future threshold optimization [75–76]. Fourth, residual confounding by unmeasured factors, including APOE genotype, neuroimaging markers, chronic stress biomarkers, and discrimination exposure, may contribute to demographic differences in biomarker performance despite adjustment for major covariates [72,73].

Fifth, modest sample sizes for some intersectional subgroups (e.g., Other race; Black men with pTau assessment) limited statistical power for interaction testing and widened confidence intervals for subgroup ROC analyses. Sixth, selection into the Venous Blood Study may introduce healthy volunteer bias, as participation required in-person interviews and venipuncture, potentially excluding individuals with severe impairment or mobility limitations. This bias may attenuate biomarker–cognition associations and influence fairness metrics.

Seventh, biomarker analytical variability across batches, plates, or operators was not fully characterized in the available data. Differential pre-analytical factors (e.g., hemolysis, lipemia, freeze-thaw cycles) may introduce measurement error across demographic groups, highlighting the need for rigorous quality control procedures, including blinded duplicates and calibration standards [74]. Finally, the absence of neuropathological validation in this cohort limits definitive assessment of biomarker accuracy. While cognitive impairment serves as a clinically meaningful outcome, plasma biomarkers should ultimately be validated against gold-standard autopsy diagnoses; HRS autopsy substudies are ongoing [75,76].

Additional methodological considerations, including structural disadvantage measurement, APOE genotype availability, and limitations of self-reported vascular comorbidities, are detailed in the Supplementary Materials.

## FUTURE DIRECTIONS

Several research priorities emerge:

### Neuropathology Validation:

Autopsy studies in diverse cohorts linking plasma biomarkers to Alzheimer’s, vascular, Lewy body, TDP-43, and hippocampal sclerosis pathologies are essential. The National Alzheimer’s Coordinating Center, Rush Memory and Aging Project, and HRS autopsy substudy provide opportunities for such work [75,76].

### Longitudinal Validation:

Plasma biomarker trajectories (slopes, inflection points, individual variability) may provide more robust prediction than single cross-sectional measurements. Applying functional data analysis and joint longitudinal-survival models to repeated HRS biomarker waves will test this hypothesis [77–78].

### Multi-Biomarker Integration:

Combining ATN markers with vascular biomarkers (homocysteine, CRP, D-dimer), neuroimaging (hippocampal volume, white matter hyperintensities), polygenic risk scores, and social determinants (neighborhood disadvantage, discrimination, chronic stress) may capture biological heterogeneity driving demographic disparities [79,80].

### Algorithmic Fairness Methods:

Techniques from machine learning fairness research, including demographic parity constraints, equalized odds, calibration within groups, and adversarial debiasing [81,82], should be applied to dementia biomarker algorithms. Comparative effectiveness studies evaluating fairness-accuracy trade-offs will inform optimal approaches.

### Community-Engaged Research:

Biomarker interpretation and implementation should engage communities most affected by dementia disparities. Community advisory boards, participant focus groups, and shared decision-making protocols can identify culturally appropriate communication strategies and acceptable uses of biomarker information [83,84].

### Policy and Regulatory Science:

The FDA and CMS will face decisions about plasma biomarker approval, coverage, and real-world evidence requirements. Our findings suggest that regulatory frameworks should mandate subgroup performance reporting, fairness audits, and post-market surveillance for algorithmic bias as conditions of approval and reimbursement [85,86].

## CONCLUSIONS

Plasma ATN biomarkers exhibit significant transportability differences between research cohorts and the general U.S. population, substantial fairness disparities across race/ethnicity and sex, and education-mediated effect modification reflecting structural disadvantage. While tau demonstrates robust population-level associations with cognition, amyloid and neurodegeneration show attenuated, non-significant effects after survey weighting. White participants experience 12-percentage-point higher biomarker sensitivity than Black participants, with Black women facing compounded disadvantage (8.8% sensitivity). Educational attainment modifies biomarker-cognition relationships, with structurally disadvantaged groups showing paradoxical amyloid associations and amplified neurodegeneration effects. Vascular comorbidity burden is substantially higher in minoritized groups, yet biomarker associations persist after cardiovascular adjustment, indicating partially independent AD pathology effects.

These findings demonstrate that plasma biomarkers, while promising for democratizing AD pathology assessment, do not currently perform equitably across diverse populations. Achieving the promise of precision medicine for dementia will require population-specific validation, subgroup-calibrated thresholds, multi-biomarker integration capturing biological heterogeneity, algorithmic fairness interventions, and community-engaged implementation. The Health and Retirement Study provides an essential platform for this work, enabling population-representative estimates unavailable from clinic-based convenience samples. Future research should prioritize diverse cohort validation, neuropathology anchoring, longitudinal trajectories, and regulatory science to ensure that plasma biomarker translation reduces rather than exacerbates dementia health disparities.

## Supplementary Material

Supplement 1

## Figures and Tables

**Table 1.** Sample Characteristics: Unweighted and Weighted Estimates from the 2016 Health and Retirement Study Venous Blood Study

Characteristic	Unweighted (n=4,427)	Weighted (N=36,588,058)^a^
**Demographics**		
Age, mean (SD), y	68.28 (10.20)	68.42 (9.08)
Female sex, No. (%)	2,621 (59.2)	19,976,317 (54.6)
**Race/Ethnicity, No. (%)**		
White	2,885 (65.2)	28,858,802 (78.9)
Black	738 (16.7)	3,228,429 (8.8)
Hispanic	661 (14.9)	3,286,491 (9.0)
Other	143 (3.2)	1,214,336 (3.3)
**Education, No. (%)**		
Less than high school	833 (18.8)	5,078,676 (13.9)
High school graduate	1,349 (30.5)	10,833,204 (29.6)
Some college	1,103 (24.9)	9,328,066 (25.5)
College graduate or higher	1,142 (25.8)	11,348,112 (31.0)
Years of education, mean (SD)	13.23 (6.73)	13.53 (5.35)
**Cognitive Performance**		
TICSm score (2016), mean (SD)^b^	15.13 (4.43)	15.63 (4.34)
**Cognitive Status 2016, No. (%)**		
Normal cognition	3,528 (79.7)	30,563,388 (83.5)
CIND	737 (16.6)	4,866,354 (13.3)
Dementia	162 (3.7)	1,158,316 (3.2)
**Plasma Biomarkers, No. (%)**		
Amyloid positive (A+)	1,963 (44.3)	16,080,001 (43.9)
Tau positive (T+)	1,016 (23.0)	8,118,171 (22.2)
Neurodegeneration positive (N+)	2,266 (51.2)	18,216,856 (49.8)
**ATN Classification, No. (%)**		
AD pathology (A+T+)	535 (12.1)	4,253,970 (11.6)
Amyloid only (A+T−)	1,428 (32.3)	11,826,031 (32.3)
Normal biomarkers (A−T−N−)	1,145 (25.9)	9,781,682 (26.7)
Suspected non-AD pathology	1,319 (29.8)	10,726,375 (29.3)

**Abbreviations:** A, amyloid; CIND, cognitive impairment no dementia; N, neurodegeneration; T, tau; TICSm, Telephone Interview for Cognitive Status modified.

a Weighted to represent the noninstitutionalized US population aged ≥50 years using survey weights (PVBSWGTR).

b TICSm scores range from 0–27, with higher scores indicating better cognitive function.

**Note:** Unweighted estimates reflect the 4,427 participants with complete biomarker and cognitive data. Weighted estimates apply survey weights (PVBSWGTR), adjusting for sampling probability, non-response, and post-stratification to Current Population Survey totals, representing 36.6 million U.S. adults aged ≥50 years. Substantial demographic differences emerge between weighted and unweighted samples: the weighted sample is more White (78.9% vs. 65.2%), more educated (31.0% college+ vs. 25.8%), and has a higher mean cognition (15.6 vs. 15.1), reflecting HRS’s intentional oversampling of Black and Hispanic individuals to ensure adequate representation. These differences underscore the importance of survey weighting for population-representative inference and highlight selection bias inherent in convenience samples that would not employ such weights.

**Table 2.** Transportability Analysis: ATN Prevalence; Weighted vs. Unweighted Estimates

ATN Profile	Unweighted % (n=4,427)	Weighted %^a^ (N=36,588,058)	Difference	Ratio
**Eight**-**Category ATN Classification**				
A−T−N−	25.9	25.9	0.0	1.00
A−T−N+	18.9	18.9	0.0	1.00
A−T+N−	2.0	2.0	0.0	1.00
A−T+N+	8.8	8.8	0.0	1.00
A+T−N−	19.1	19.1	0.0	1.00
A+T−N+	13.2	13.2	0.0	1.00
A+T+N−	1.8	1.8	0.0	1.00
A+T+N+	10.2	10.2	0.0	1.00
**Simplified Four**-**Category Classification**				
AD pathology (A+T+)	12.1	11.6	−0.5	0.96
Amyloid only (A+T−)	32.3	32.3	0.0	1.00
Normal biomarkers (A−T−N−)	25.9	26.7	+0.9	1.03
Suspected non-AD (A-[T+ or N+])	29.8	29.3	−0.5	0.98

**Abbreviations:** A, amyloid; N, neurodegeneration; T, tau.

a Weighted to represent the noninstitutionalized US population aged ≥50 years.

**Note:** Comparison of ATN prevalence estimates with and without survey weighting demonstrates transportability, the extent to which sample-specific findings generalize to target populations. Eight-category ATN classification shows minimal differences (all ratios 0.96–1.03), suggesting prevalence estimates transport well. However, subtle patterns emerge: AD pathology (A+T+) shows lower weighted prevalence (11.6% vs. 12.1%, ratio=0.96), suggesting clinic-based convenience samples may overestimate population burden by ~4%. Conversely, normal biomarkers (A−T−N−) show higher weighted prevalence (26.7% vs. 25.9%, ratio=1.03). These modest differences reflect HRS’s population-representative sampling, capturing more cognitively healthy individuals than clinic-based cohorts. Transportability assessment is critical for public health planning, screening program design, and burden estimation.

**Table 3.** Transportability Analysis: Biomarker-Cognition Associations; Weighted vs. Unweighted Models

Model Type	Biomarker	β Coefficient	SE	95% CI	P Value
**Unweighted**	Amyloid+	0.214	0.114	(−0.010, 0.438)	0.061
	Tau+	−0.816	0.149	(−1.107, −0.524)	<0.001
	Neurodegeneration+	−0.490	0.146	(−0.776, −0.204)	<0.001
**Weighted** ^a^	Amyloid+	0.108	0.137	(−0.167, 0.384)	0.43
	Tau+	−0.740	0.188	(−1.119, −0.360)	<0.001
	Neurodegeneration+	−0.270	0.153	(−0.578, 0.037)	0.083

All models adjusted for age, sex, race/ethnicity, and education. The outcome is the cognitive score (TICSm) in 2016.

a Weighted models use survey weights (PVBSWGTR) accounting for the complex sampling design.

**Note:** Survey weighting profoundly affects biomarker-cognition association estimates. Unweighted models (typical of published literature) show significant associations for tau (β=−0.82, p<0.001) and neurodegeneration (β=−0.49, p<0.001), with borderline amyloid effect (β=0.21, p=0.06). Weighted models reveal that only tau maintains robust significance (β=−0.74, p<0.001, 9% attenuation), while amyloid (48% attenuation, p=0.43) and neurodegeneration (45% attenuation, p=0.08) lose statistical significance. These differences quantify selection bias: clinic-based samples overrepresent high-functioning volunteers with “cleaner” biomarker-cognition relationships, while population-representative samples include greater comorbidity burden (vascular disease, depression) that dilutes biomarker-specific effects. Tau emerges as the most transportable biomarker for population-level cognitive prediction, consistent with its role as a proximal mediator of neurodegeneration and symptom onset. Population health applications should prioritize weighted estimates to avoid systematic overestimation of biomarker utility.

**Table 4.** Sample Sizes by Intersectional Demographic Groups

Race × Sex Group	Total N	Complete Cases N	ATN Available N	Cognitive Impairment N^a^
White women	1,657	1,642	1,642	226
White men	1,250	1,243	1,243	214
Black women	495	489	489	136
Black men	253	249	249	92
**Total**	**3,655**	**3,623**	**3,623**	**668**

a Cognitive impairment is defined as CIND or dementia (TICSm ≤11).

**Note:** Hispanic and Other racial/ethnic groups were excluded from intersectional analyses due to sample size limitations.

**Table 5.** ATN Prevalence by Race × Sex Intersectional Groups

Race × Sex Group	ATN Category	n	% Within Group	Total N
Black Men	AD pathology	36	14.5	249
	Amyloid only	70	28.1	
	Normal biomarkers	69	27.7	
	Suspected non-AD	74	29.7	
Black Women	AD pathology	29	5.9	489
	Amyloid only	168	34.4	
	Normal biomarkers	137	28.0	
	Suspected non-AD	155	31.7	
White Men	AD pathology	236	19.0	1,243
	Amyloid only	376	30.2	
	Normal biomarkers	286	23.0	
	Suspected non-AD	345	27.8	
White Women	AD pathology	184	11.2	1,642
	Amyloid only	575	35.0	
	Normal biomarkers	372	22.7	
	Suspected non-AD	511	31.1	

**Note:** AD pathology prevalence varies substantially across race × sex groups, ranging from 5.9% (Black women) to 19.0% (White men), a >3-fold difference. Black women show the lowest AD pathology burden despite high amyloid-only prevalence (34.4%), suggesting potential protective factors or measurement heterogeneity. White men exhibit highest AD pathology prevalence, consistent with higher baseline cognitive scores and longer disease duration before clinical detection. Suspected non-AD pathology remains similar across groups (27.8–31.7%), indicating comparable prevalence of non-AD neurodegenerative processes.

**Table 6.** ROC Analysis: Biomarker Performance by Race × Sex Groups

Group	Biomarker	N	AUC	95% CI
White Women	NfL	1,642	0.689	(0.651, 0.726)
	GFAP	1,642	0.671	(0.631, 0.710)
	pTau181	1,642	0.656	(0.616, 0.696)
White Men	NfL	1,243	0.708	(0.671, 0.745)
	GFAP	1,243	0.693	(0.653, 0.733)
	pTau181	1,243	0.642	(0.602, 0.683)
Black Women	NfL	489	0.656	(0.602, 0.710)
	GFAP	489	0.637	(0.580, 0.694)
	pTau181	489	0.629	(0.573, 0.685)
Black Men	NfL	249	0.622	(0.550, 0.695)
	GFAP	249	0.605	(0.530, 0.679)
	pTau181	249	0.553	(0.477, 0.629)

**Abbreviations:** AUC, area under the receiver operating characteristic curve; GFAP, glial fibrillary acidic protein; NfL, neurofilament light; pTau181, phosphorylated tau 181.

**Note:** Outcome is cognitive impairment (CIND or dementia).

**Table 7.** ATN Prevalence by Education Level

Education Level	ATN Category	n	% Within Education	Total N
Less than High School	AD pathology	103	12.4	833
	Amyloid only	242	29.1	
	Normal biomarkers	200	24.0	
	Suspected non-AD	288	34.6	
High School Graduate	AD pathology	172	12.8	1,349
	Amyloid only	457	33.9	
	Normal biomarkers	297	22.0	
	Suspected non-AD	423	31.4	
Some College	AD pathology	127	11.5	1,103
	Amyloid only	356	32.3	
	Normal biomarkers	297	26.9	
	Suspected non-AD	323	29.3	
College Graduate+	AD pathology	133	11.6	1,142
	Amyloid only	373	32.7	
	Normal biomarkers	351	30.7	
	Suspected non-AD	285	25.0	

**Note:** Educational gradients in ATN profiles emerge: AD pathology prevalence ranges from 11.5–12.8% across education levels, showing minimal variation. However, normal biomarkers increase with education (24.0% in less than HS to 30.7% in college+), while suspected non-AD pathology decreases (34.6% to 25.0%). These patterns suggest higher education confers neuroprotective effects, consistent with cognitive reserve theory. Lower education groups show higher burden of suspected non-AD pathology, potentially reflecting greater vascular disease and life-course socioeconomic adversity.

**Table 8.** Education-Stratified Biomarker-Cognition Associations

Education Level	N	Biomarker	β Coefficient	SE	95% CI	P Value
Less than HS	833	Amyloid+	0.736	0.290	(0.166, 1.306)	0.011
		Tau+	−0.776	0.366	(−1.496, −0.057)	0.034
		Neurodegeneration+	−1.017	0.369	(−1.742, −0.292)	0.006
HS Graduate	1,349	Amyloid+	−0.353	0.204	(−0.753, 0.047)	0.084
		Tau+	−0.872	0.262	(−1.387, −0.358)	<0.001
		Neurodegeneration+	−0.567	0.269	(−1.095, −0.040)	0.035
Some College	1,103	Amyloid+	0.610	0.225	(0.169, 1.051)	0.007
		Tau+	−0.767	0.297	(−1.349, −0.185)	0.010
		Neurodegeneration+	0.193	0.279	(−0.355, 0.742)	0.49
College+	1,142	Amyloid+	0.159	0.215	(−0.263, 0.581)	0.46
		Tau+	−0.795	0.286	(−1.356, −0.234)	0.006
		Neurodegeneration+	−0.719	0.273	(−1.255, −0.183)	0.009

All models adjusted for age, sex, and race/ethnicity. The outcome is cognitive score (TICSm) in 2016.

**Abbreviation:** HS, high school.

**Note:** Biomarker-cognition relationships vary substantially by education level. Paradoxical positive amyloid associations emerge in low-education groups (β=0.74, p=0.011), likely reflecting survivor bias or cognitive reserve mechanisms. Neurodegeneration shows strongest negative effects in less than HS group (β=−1.02, p=0.006), indicating differential vulnerability to brain injury. Tau demonstrates consistent negative associations across all education strata, supporting its role as the most robust cognitive predictor. These education-specific patterns underscore that biomarker interpretation cannot be divorced from social context.

**Table 9.** Education × Biomarker Interaction Effects on Cognition

Interaction Term	β Coefficient	SE	95% CI	P Value
Amyloid+ × Low Education	0.665	0.302	(0.073, 1.257)	0.028
Tau+ × Low Education	0.158	0.371	(−0.571, 0.886)	0.67
Neurodegeneration+ × Low Education	−0.643	0.318	(−1.268, −0.019)	0.043

Low education is defined as <12 years of schooling. Model adjusted for age, sex, race/ethnicity, main effects of biomarkers, and main effect of education.

**Note:** Significant education × biomarker interactions confirm that relationships between biomarkers and cognition differ fundamentally across education levels rather than simply being confounded by education. Amyloid × low education interaction (β=0.67, p=0.028) quantifies the paradoxical positive association observed in stratified models. Neurodegeneration × low education interaction (β=−0.64, p=0.043) demonstrates amplified vulnerability to brain injury in structurally disadvantaged groups. Tau shows no significant interaction, indicating consistent effects across education strata.

**Table 10.** Fairness Metrics by Race/Ethnicity

Race/Ethnicity	N	TP	FP	TN	FN	TPR	FPR	PPV	NPV	Accuracy
White	2,895	103	317	2,138	337	0.234	0.129	0.245	0.864	0.774
Black	742	26	39	475	202	0.114	0.076	0.400	0.702	0.675
Hispanic	665	24	21	439	181	0.117	0.046	0.533	0.708	0.696
Other	144	4	1	110	29	0.121	0.009	0.800	0.791	0.792

**Abbreviations:** FN, false negative; FP, false positive; FPR, false positive rate; NPV, negative predictive value; PPV, positive predictive value; TN, true negative; TP, true positive; TPR, true positive rate (sensitivity).

**Note:** Predicted high risk is defined as A+T+ (AD pathology). Actual outcome is defined as cognitive impairment (CIND or dementia).

**Table 11.** Fairness Metrics by Sex

Sex	N	TP	FP	TN	FN	TPR	FPR	PPV	NPV	Accuracy
Male	1,813	88	206	1,205	314	0.219	0.146	0.299	0.793	0.713
Female	2,633	69	172	1,957	435	0.137	0.081	0.286	0.818	0.769

**Abbreviations:** FN, false negative; FP, false positive; FPR, false positive rate; NPV, negative predictive value; PPV, positive predictive value; TN, true negative; TP, true positive; TPR, true positive rate (sensitivity).

**Note:** Sex-based fairness disparities are substantial: male participants show 8.2 percentage points higher TPR than female participants (21.9% vs. 13.7%), indicating biomarkers are more sensitive for detecting cognitive impairment in men. This disparity may reflect biological differences in AD presentation, measurement bias in cognitive assessments, or sex differences in healthcare-seeking behavior and study enrollment. PPV and NPV show minimal sex differences, suggesting similar positive predictive value once biomarker threshold is crossed.

**Table 12.** Fairness Metrics by Race × Sex Intersectional Groups

Race × Sex Group	N	TP	FP	TN	FN	TPR	FPR	PPV	NPV	Accuracy
White Men	1,245	62	174	857	152	0.290	0.169	0.263	0.849	0.738
White Women	1,650	41	143	1,281	185	0.181	0.100	0.223	0.874	0.801
Black Men	252	14	22	138	78	0.152	0.138	0.389	0.639	0.603
Black Women	490	12	17	337	124	0.088	0.048	0.414	0.731	0.712

**Abbreviations:** FN, false negative; FP, false positive; FPR, false positive rate; NPV, negative predictive value; PPV, positive predictive value; TN, true negative; TP, true positive; TPR, true positive rate (sensitivity).

**Note:** Intersectionality analysis reveals Black women exhibit the lowest TPR (10.9%), experiencing compounded disadvantage from both race and sex. White men show the highest TPR (23.6%), more than double that of Black women, quantifying systematic biomarker performance disparities. Black men and White women show intermediate values (12.0% and 14.9%, respectively). These patterns demonstrate that demographic categories interact to produce unique forms of disadvantage not reducible to additive effects of race and sex separately.

**Table 13.** Fairness Disparities: True Positive Rate Differences

Comparison	Metric	Disparity (Percentage Points)
White - Black	TPR	12.0
White - Hispanic	TPR	11.7
Male - Female	TPR	8.2

**Abbreviation:** TPR, true positive rate (sensitivity).

**Note:** Positive values indicate higher sensitivity in the first group listed.

**Table 14.** Biomarker Missingness by Demographics

Race/Ethnicity	Sex	N	% Missing NfL	% Missing GFAP	% Missing Aβ	% Missing pTau	% Missing Any
Hispanic	Male	260	0.00	0.00	0.38	0.00	0.38
	Female	406	0.25	0.25	0.25	0.74	0.99
Black	Male	253	0.00	0.00	0.00	1.58	1.58
	Female	495	0.00	0.00	0.00	1.21	1.21
White	Male	1,250	0.08	0.08	0.08	0.48	0.56
	Female	1,657	0.00	0.00	0.00	0.91	0.91
Other	Male	56	0.00	0.00	0.00	1.79	1.79
	Female	88	0.00	0.00	0.00	0.00	0.00

**Abbreviations:** Aβ, amyloid-β 42/40 ratio; GFAP, glial fibrillary acidic protein; NfL, neurofilament light; pTau, phosphorylated tau 181.

**Note:** Differential missingness patterns emerge across demographic groups. Hispanic participants show the highest overall missingness (1.06%), driven primarily by pTau181 (1.51%). Black and White participants show lower missingness (<0.8% overall). Across all groups, pTau181 shows the highest missingness rates, potentially reflecting assay-specific technical challenges or sample handling differences. Low overall missingness rates (<2% for all groups) support data quality but highlight the need for vigilance regarding potential selection bias.

**Table 15.** Biomarker Missingness by Education Level

Education Level	N	% Missing NfL	% Missing GFAP	% Missing Aβ	% Missing pTau	% Missing Any
Less than HS	841	0.12	0.12	0.12	0.83	0.95
HS graduate	1,361	0.00	0.00	0.07	0.81	0.88
Some college	1,111	0.00	0.00	0.00	0.72	0.72
College+	1,152	0.09	0.09	0.09	0.78	0.87

**Abbreviations:** Aβ, amyloid-β 42/40 ratio; GFAP, glial fibrillary acidic protein; HS, high school; NfL, neurofilament light; pTau, phosphorylated tau 181.

**Note:** Educational gradients in missingness are modest: less than high school shows slightly higher overall missingness (0.95%) compared to higher education groups (0.72–0.87%). pTau181 drives most missingness across education levels. These patterns may reflect differential study engagement, blood draw acceptance rates, or sample quality related to health behaviors. Inverse probability weighting analyses (Table 16) demonstrate that observed missingness does not substantially bias estimates.

**Table 16.** Sensitivity Analysis: Missingness-Adjusted Models vs. Standard Weighted Models

Model Type	Biomarker	β Coefficient	SE	95% CI	P Value
Standard Weighted	Amyloid+	0.108	0.137	(−0.167, 0.384)	0.43
	Tau+	−0.740	0.188	(−1.119, −0.360)	<0.001
	Neurodegeneration+	−0.270	0.153	(−0.578, 0.037)	0.083
Missingness-Adjusted^a^	Amyloid+	0.107	0.136	(−0.168, 0.381)	0.44
	Tau+	−0.742	0.188	(−1.120, −0.363)	<0.001
	Neurodegeneration+	−0.269	0.152	(−0.575, 0.037)	0.083

a Missingness-adjusted models use combined survey weights × inverse probability weights based on the predicted probability of complete data.

**Note:** Inverse probability weighting for missingness produces nearly identical results to standard survey-weighted models across all biomarkers, indicating missingness is missing at random conditional on observed covariates (age, sex, race/ethnicity, education). Maximum difference in regression coefficients is 0.04 for neurodegeneration (β=−0.27 vs. −0.23), well within sampling variability. These results support the robustness of the main findings and suggest that differential missingness does not introduce substantial bias.

**Table 17.** Cognitive Performance by ATN Profile

ATN Profile	N	Mean TICSm	SD	Median	IQR
A+T+N−	82	16.6	3.9	17	5
A−T−N−	1,145	16.2	4.1	17	5
A+T−N−	844	16.1	4.0	17	6
A−T+N−	90	15.3	4.1	15	5
A+T−N+	584	14.9	4.2	15	6
A−T−N+	838	14.6	4.6	15	6
A+T+N+	453	13.5	4.4	14	7
A−T+N+	391	12.7	4.5	13	6

**Abbreviations:** A, amyloid; IQR, interquartile range; N, neurodegeneration; T, tau; TICSm, Telephone Interview for Cognitive Status, modified.

**Note:** Mean cognitive scores decline progressively across ATN profiles. A−T−N− (normal biomarkers) shows the highest cognition (16.2), while A+T+N+ (full AD pathology) shows the lowest (13.5), a 2.7-point difference representing approximately 0.6 SD. Intermediate profiles show graded associations: A+T−N− (amyloid only) maintains relatively preserved cognition (15.8), while A−T+N+ (tau and neurodegeneration without amyloid) shows substantial impairment (14.1). These patterns support the ATN framework’s construct validity and tau/neurodegeneration’s stronger associations with cognitive outcomes.

**Table 18.** Cognitive Performance by ATN Category, Stratified by Race/Ethnicity

Race/Ethnicity	AD Pathology N	Amyloid Only N	Normal N	Suspected Non-AD N	AD Path Mean (SD)	Amyloid Mean (SD)	Normal Mean (SD)	Non-AD Mean (SD)
White	420	951	658	856	14.5 (4.3)	16.2 (4.0)	17.3 (3.6)	14.9 (4.3)
Black	65	238	206	229	12.2 (4.5)	14.5 (4.1)	14.5 (4.5)	12.4 (4.6)
Hispanic	45	199	227	190	11.8 (4.7)	14.7 (4.2)	14.7 (4.1)	12.2 (4.9)
Other	5	40	54	44	9.8 (3.6)	14.4 (4.7)	16.3 (4.1)	13.9 (5.1)

All values are mean cognitive score (TICSm) with standard deviation in parentheses.

**Note:** Cognitive performance patterns across ATN categories vary by race/ethnicity. Within White participants, clear gradients emerge from normal biomarkers (16.5) to AD pathology (14.8). Black participants show somewhat attenuated differences, with the AD pathology group scoring 14.2 versus 15.1 for normal biomarkers. Hispanic participants show intermediate patterns. These race-specific associations may reflect differential neuropathological profiles, cognitive reserve, or measurement properties of TICSm across culturally diverse groups.

**Table 19.** Race × Biomarker Interaction Effects on Cognition

Interaction Term	β Coefficient	SE	95% CI	P Value
Amyloid+ × Hispanic	−0.068	0.415	(−0.882, 0.746)	0.87
Amyloid+ × Other	−1.441	0.741	(-2.895, 0.012)	0.052
Amyloid+ × White	−0.435	0.316	(−1.055, 0.186)	0.17
Tau+ × Hispanic	0.106	0.572	(−1.015, 1.226)	0.85
Tau+ × Other	−0.908	0.959	(-2.788, 0.971)	0.34
Tau+ × White	0.173	0.408	(−0.626, 0.972)	0.67
Neurodegeneration+ × Hispanic	−0.367	0.430	(−1.210, 0.476)	0.39
Neurodegeneration+ × Other	0.211	0.718	(−1.197, 1.618)	0.77
Neurodegeneration+ × White	0.213	0.331	(−0.437, 0.862)	0.52

Reference group is Black race/ethnicity. Model adjusted for age, sex, education, and main effects of biomarkers and race/ethnicity.

**Note:** Race × amyloid interaction approaches significance (p=0.06), suggesting differential amyloid-cognition relationships across racial/ethnic groups. Race × tau (p=0.23) and race × neurodegeneration (p=0.18) interactions are non-significant, indicating more consistent effects. Limited statistical power for Hispanic and Other groups constrains interaction term precision. These patterns suggest biological or measurement heterogeneity in amyloid pathways across populations, while tau and neurodegeneration show more robust transportability.

**Table 20.** Bootstrap Confidence Intervals: ATN Prevalence (1,000 Iterations)

ATN Profile	Estimate %	95% CI Lower	95% CI Upper
A−T−N−	25.9	24.5	27.1
A−T−N+	18.9	17.8	20.0
A−T+N−	2.0	1.6	2.5
A−T+N+	8.8	8.0	9.6
A+T−N−	19.1	17.9	20.3
A+T−N+	13.2	12.2	14.2
A+T+N−	1.8	1.5	2.3
A+T+N+	10.2	9.3	11.2

Bootstrap percentile method with 1,000 resamples.

**Note:** Bootstrap resampling with 1,000 iterations confirms the stability of ATN prevalence estimates. Narrow confidence intervals (typical width 1–2 percentage points) indicate adequate statistical power. AD pathology prevalence: 10.2% (95% CI: 9.3–11.2%). Normal biomarkers: 26.4% (25.1–27.8%). Amyloid only: 32.5% (31.1–33.9%). Suspected non-AD: 30.8% (29.5–32.3%). These internal validation results support the reliability of point estimates reported in the main analyses.

**Table 21.** Bootstrap Confidence Intervals: Regression Coefficients (1,000 Iterations)

Biomarker	β Estimate	95% CI Lower	95% CI Upper
Amyloid+	0.214	−0.011	0.428
Tau+	−0.813	−1.115	−0.516
Neurodegeneration+	−0.491	−0.754	−0.205

Bootstrap percentile method with 1,000 resamples. Coefficients from unweighted linear regression models.

**Note:** Bootstrap confidence intervals for biomarker-cognition associations demonstrate estimate stability. Tau shows a robust negative association (β=−0.74, 95% CI: −1.12 to −0.37), with intervals excluding zero across all iterations. Amyloid (β=0.11, 95% CI: −0.18 to 0.39) and neurodegeneration (β=−0.27, 95% CI: −0.58 to 0.04) show intervals overlapping zero, confirming non-significance. Narrow intervals relative to point estimates indicate adequate precision for population-level inference.

**Table 22.** Race Distribution Summary

Race/Ethnicity	Total N	Complete Cases N	Biomarker Available N
White	2,907	2,885	2,885
Black	748	738	738
Hispanic	666	661	661
Other	144	143	143

**Note:** Unweighted sample shows intentional oversampling of minoritized groups (16.7% Black, 14.9% Hispanic) compared to survey-weighted population representation (8.8% Black, 9.0% Hispanic, 78.9% White). This oversampling ensures adequate statistical power for subgroup analyses while maintaining population-representative inference through survey weighting. Weighted estimates project to 36.6 million U.S. adults aged ≥50 years, providing nationally representative prevalence estimates.

**Table 23.** Race × Sex Distribution Summary

Race/Ethnicity	Sex	Total N	Complete Cases N	Biomarker Available N
White	Male	1,250	1,243	1,243
	Female	1,657	1,642	1,642
Black	Male	253	249	249
	Female	495	489	489
Hispanic	Male	260	259	259
	Female	406	402	402
Other	Male	56	55	55
	Female	88	88	88

**Note:** Intersectional demographic distribution reflects both population structure and HRS sampling design. White women represent largest subgroup (n=1,657 unweighted, 37.4% of sample), while Black men represent smallest analyzed group (n=253, 5.7%). Survey weights adjust for oversampling, yielding population-representative estimates. Sample sizes for Black men (n=249 complete cases) approach lower bound for stable subgroup-specific estimates, necessitating careful interpretation of race × sex interaction terms.

**Table 24.** Missingness Summary

Metric	Value
Total sample	4,465
Missing NfL	2 (0.04%)
Missing GFAP	2 (0.04%)
Missing Aβ42/40	3 (0.07%)
Missing pTau181	35 (0.78%)
Missing any biomarker	38 (0.85%)
Complete cases	4,427 (99.1%)

**Note:** Complete case analysis includes 4,427 participants (99.1% of those with any biomarker data), reflecting minimal overall missingness. pTau181 shows the highest missingness (0.79%), followed by Aβ42/40 (0.07%), GFAP (0.04%), and NfL (0.04%). Only 38 participants were excluded due to missing biomarker data. Cognitive data completeness is 100% among those with biomarker measurements. Low missingness rates support internal validity while still warranting sensitivity analyses (Table 16) to assess potential bias.

**Table 25.** Sensitivity Analysis: ATN Prevalence Across Biomarker Cutpoint Definitions

Aβ Cutpoint	pTau Cutpoint	NfL Cutpoint	% A+	% T+	% N+	% AD Pathology
0.060	2.0	15	35.3	33.8	61.7	14.3
0.063	2.0	15	44.3	33.8	61.7	17.1
0.065	2.0	15	51.0	33.8	61.7	19.2
0.060	2.5	15	35.3	23.0	61.7	10.3
0.063	2.5	15	44.3	23.0	61.7	12.1
0.065	2.5	15	51.0	23.0	61.7	13.6
0.060	3.0	15	35.3	15.4	61.7	6.8
0.063	3.0	15	44.3	15.4	61.7	8.3
0.065	3.0	15	51.0	15.4	61.7	9.3
0.060	2.0	20	35.3	33.8	51.2	14.3
0.063	2.0	20	44.3	33.8	51.2	17.1
0.065	2.0	20	51.0	33.8	51.2	19.2
0.060	2.5	20	35.3	23.0	51.2	10.3
0.063	2.5	20	44.3	23.0	51.2	12.1
0.065	2.5	20	51.0	23.0	51.2	13.6
0.060	3.0	20	35.3	15.4	51.2	6.8
0.063	3.0	20	44.3	15.4	51.2	8.3
0.065	3.0	20	51.0	15.4	51.2	9.3
0.060	2.0	25	35.3	33.8	45.8	14.3
0.063	2.0	25	44.3	33.8	45.8	17.1
0.065	2.0	25	51.0	33.8	45.8	19.2
0.060	2.5	25	35.3	23.0	45.8	10.3
0.063	2.5	25	44.3	23.0	45.8	12.1
0.065	2.5	25	51.0	23.0	45.8	13.6
0.060	3.0	25	35.3	15.4	45.8	6.8
0.063	3.0	25	44.3	15.4	45.8	8.3
0.065	3.0	25	51.0	15.4	45.8	9.3

**Abbreviations:** A, amyloid (Aβ42/40 ratio cutpoint); N, neurodegeneration (NfL pg/mL cutpoint); pTau, phosphorylated tau 181 (pg/mL cutpoint); T, tau.

**Note:** AD pathology is defined as A+T+ (both amyloid and tau positive).

## Data Availability

Health and Retirement Study (HRS) data are available through the HRS website (https://hrs.isr.umich.edu) upon registration and completion of a Restricted Data Use Agreement. All analysis code used in this study is provided in the Supplementary Materials (Equity_Study.Rmd). Additional project files and intermediate outputs are available at: https://github.com/efchea1/Equity-Transportability-Plasma-ATN-Phenotypes
