# Supplementary material for: Equity and Transportability of Plasma ATN Phenotypes in a Population-Representative U.S. Aging Cohort
Source: medRxiv. 2026 Feb 5:2026.01.31.26344775. Preprint. [Version 4] doi: 10.64898/2026.01.31.26344775 (PMC12889772; doi:10.64898/2026.01.31.26344775)
Supplement: Supplementary file 1 [file media-1.pdf]

SUPPLEMENTARY MATERIALS

SUPPLEMENTARY METHODS

Survey Weight Construction and Application

HRS employs a complex multistage probability sampling design with stratification by geography and race/ethnicity, clustering within primary sampling units, and oversampling of Black and Hispanic individuals. Person-level survey weights (PVBSWGTR) adjust for: (1) base sampling probability; (2) non-response at household and individual levels; (3) post-stratification to Current Population Survey marginal totals for age, sex, and race/ethnicity.

We applied survey weights using the R survey package [20], specifying:

- **Weights:** PVBSWGTR
- **Strata:** STRATUM
- **Primary sampling units:** SECU
- **Nesting:** TRUE (to accommodate certainty PSUs)

All prevalence estimates, means, regression coefficients, and confidence intervals presented in the main manuscript reflect survey-weighted analyses unless explicitly noted as "unweighted."

Inverse Probability Weighting for Missingness

To assess whether differential missingness biased estimates, we constructed inverse probability weights (IPW) as follows:

1. Fitted logistic regression predicting complete-case status (all biomarkers and cognition observed) from age, sex, race/ethnicity, and education.
2. Predicted probability of being a complete case for all individuals: $\widehat{p}_i = P(Complete \mid X_i$
3. Calculated IPW: $w_i^{IPW} = \frac{1}{\widehat{p}_i}$ for complete cases, 0 otherwise.
4. Combined with survey weights: $w_i^{combined} = w_i^{survey} \times w_i^{IPW}$
5. Truncated at the 99th percentile to reduce extreme weight influence.

Models using combined weights produced nearly identical results to standard survey-weighted models (Table 16), indicating that missingness was missing at random conditional on observed covariates.

Bootstrap Internal Validation

To assess internal validity and quantify sampling variability, we conducted 1,000 bootstrap resamples

with replacement from the complete-case analytic sample. For each resample, we calculated:

- ATN prevalence for all eight profiles
- Regression coefficients for biomarker-cognition associations

Bootstrap percentile confidence intervals (2.5th and 97.5th percentiles) are reported in Tables 20-21. Narrow intervals confirm adequate statistical power and stable estimates.

Youden Index Optimization

For each racial/ethnic group separately, we identified optimal biomarker cutpoints maximizing the Youden index:

$$J = Sensitivity + Specificity - 1$$

using the cutpointr R package. Outcome was cognitive impairment (CIND or dementia). Results are presented in Table S7 and Figure S4.

These race-specific optima provide empirical justification for considering population-specific thresholds, though ethical and practical considerations around race-specific algorithms must be weighed carefully.

SUPPLEMENTAL STRENGTHS AND LIMITATIONS

Strengths

This study's primary strength is its use of the Health and Retirement Study, the only nationally representative U.S. aging cohort with plasma AD biomarker data. The probability sampling design, oversampling of Black and Hispanic individuals, and application of survey weights enable population-level inference unavailable from clinic-based cohorts. The large sample size (n=4,427) provides adequate power for subgroup analyses while maintaining meaningful representation of structurally disadvantaged groups. Methodological rigor is enhanced through complex survey weighting, inverse probability weighting for missingness, bootstrap internal validation, calibration analyses, and extensive sensitivity analyses across biomarker thresholds. The integration of transportability, fairness, and structural disadvantage frameworks represents a novel contribution to the AD biomarker literature.

Limitations

Several methodological limitations warrant consideration. First, the cross-sectional design precludes causal inference regarding the temporal ordering of biomarker changes and cognitive decline. While biomarker-cognition associations are interpreted as reflecting underlying pathology, reverse causation or bidirectional processes cannot be excluded. Future waves of HRS biomarker data will enable longitudinal modeling of biomarker trajectories.

Second, cognitive outcomes were reconstructed from TICSm items across 2016-2020. Although validated

for population studies, TICSm is a brief screening tool with ceiling effects, limited domain coverage, and cultural measurement non-invariance. Proxy and self-reported cognition were collapsed following HRS guidelines, but proxy assessments may introduce systematic downward bias. Wave-specific differences in interview mode, particularly during the COVID-19 pandemic, may also affect measurement consistency.

Third, ATN cutpoints (Aβ42/40 <0.063, pTau181 >2.5 pg/mL, NfL >20 pg/mL) were derived from predominantly European-ancestry clinical cohorts and may not be optimal for diverse populations. Sensitivity analyses revealed >2.5-fold variation in AD pathology prevalence across plausible thresholds. Race-specific Youden-optimized cutpoints differed by up to 40%, highlighting the need for neuropathology-validated thresholds in heterogeneous populations.

Fourth, residual confounding remains possible. APOE ε4 genotype, neuroimaging markers (e.g., white matter hyperintensities), chronic stress biomarkers, discrimination exposure, and neighborhood disadvantage were unavailable but likely contribute to demographic differences in biomarker performance. Incorporating these factors in future analyses will strengthen causal inference.

Fifth, some intersectional subgroups, particularly Black men and individuals classified as "Other" race, had modest sample sizes, limiting precision for interaction models and ROC analyses. Confidence intervals for subgroup AUCs were wide, especially for pTau181 in Black men. Pooling across cohorts or leveraging future HRS biomarker waves will improve precision.

Sixth, selection into the Venous Blood Study may introduce healthy volunteer bias. Participation required in-person interviews and venipuncture, potentially excluding individuals with severe impairment, mobility limitations, or medical contraindications. Although survey weighting mitigates this bias, underrepresentation of the most impaired individuals may attenuate biomarker–cognition associations and influence fairness metrics.

Seventh, biomarker analytical variability across assay batches, plates, or operators was not fully characterized in the available data. Pre-analytical factors such as hemolysis, lipemia, and freeze-thaw cycles may vary across demographic groups and introduce differential measurement error. Rigorous quality control procedures, including blinded duplicates and calibration standards, will be essential for multi-site implementation.

Finally, the absence of neuropathological validation in this cohort limits definitive assessment of biomarker accuracy. While cognitive impairment serves as a clinically meaningful outcome, plasma biomarkers should ultimately be validated against autopsy diagnoses. Ongoing HRS autopsy substudies will enable pathology-anchored validation in future work.

SUPPLEMENTARY RESULTS

Vascular Comorbidity Prevalence by Race/Ethnicity and Sex

Vascular comorbidity burden was substantially higher in minoritized groups (Table S1). Black participants exhibited the highest prevalence of hypertension (78.2%), diabetes (35.7%), and any CVD (82.1%).

Hispanic participants showed intermediate prevalence (hypertension 64.7%, diabetes 38.1%, any CVD 73.1%). White participants had the lowest burden (hypertension 59.4%, diabetes 22.5%, any CVD 64.6%).

Within racial/ethnic groups, sex differences were modest, though Black men showed slightly higher CVD prevalence than Black women (83.7% vs. 81.3%; Table S2).

Impact of Vascular Adjustment on Biomarker-Cognition Associations

Adjusting for vascular comorbidities (hypertension, diabetes, stroke) modestly attenuated biomarker-cognition associations (Table S3; Figure S1):

- **Amyloid:** No change (β=0.11, both models)
- **Tau:** 4% attenuation (β=-0.74 → -0.71), remained highly significant (p<0.001)
- **Neurodegeneration:** 26% attenuation (β=−0.27 → -0.20), remained non-significant

These results indicate that observed biomarker-cognition relationships are not fully explained by vascular confounding, supporting partially independent AD pathology effects.

CVD-Stratified Biomarker Associations

Stratifying by CVD presence revealed that biomarker associations persist within both CVD-free and CVD-present subgroups (Table S4; Figure S2):

No CVD (n=1,341):

- Tau: β=-1.08, p<0.001
- Neurodegeneration: β=-0.29, p=0.28
- Amyloid: β=0.03, p=0.87

Any CVD (n=2,955):

- Tau: β=-0.72, p<0.001
- Neurodegeneration: β=-0.51, p=0.004
- Amyloid: β=0.27, p=0.05

Tau demonstrated robust associations regardless of vascular burden, while neurodegeneration showed stronger effects in the CVD-present group, potentially reflecting combined AD and vascular contributions to brain injury.

Calibration Analysis Details

Calibration was assessed by dividing participants into risk deciles based on predicted probability of cognitive impairment from logistic regression (A+T+N biomarkers as predictors). Within each decile and racial/ethnic group, we calculated the mean predicted risk and observed cognitive impairment rate (Table S5).

Perfect calibration corresponds to points on the 45-degree diagonal (predicted = observed). Deviations indicate systematic over- or under-prediction. Calibration statistics (Table S6) quantify model fit:

- **Calibration slope:** 1.0 indicates perfect calibration; >1 indicates overly narrow prediction intervals; <1 indicates overly wide.
- **Calibration intercept:** 0 indicates no systematic bias; <0 indicates overprediction; >0 indicates underprediction.

White participants showed near-perfect calibration (slope=0.99, intercept=-0.06), while Black and Hispanic participants showed poor calibration (slopes=1.29 and 1.78, intercepts=0.06 and −0.02), indicating that models developed in predominantly White samples do not transport accurately to minoritized populations (Figure S3).

Race-Specific Optimal Cutpoints

Youden-optimized cutpoints differed substantially by race (Table S7; Figure S4):

pTau181:

- Black: 1.54 pg/mL
- Hispanic: 1.74 pg/mL
- White: 2.16 pg/mL

NfL:

- Black: 17.6 pg/mL
- Hispanic: 21.6 pg/mL
- White: 24.0 pg/mL

GFAP:

- Black: 119.5 pg/mL
- Hispanic: 89.0 pg/mL
- White: 102.9 pg/mL

These differences (up to 40% relative) suggest that universal cutpoints may systematically misclassify minoritized individuals, contributing to observed fairness disparities. However, implementing race-specific thresholds raises ethical concerns about reifying biological race concepts and requires careful consideration of potential harms.

Fairness Landscape Across Education and Intersectional Groups

The fairness landscape heatmap (Figure S5) visualizes TPR across race × sex × education intersectional groups. Black women show persistently low TPR across all education levels, with particularly striking deficits in higher education strata:

- **Black women, less than high school:** TPR=7.9%
- **Black women, high school:** TPR=9.5%
- **Black women, some college:** TPR=5.3%
- **Black women, college+:** TPR=16.7%

In contrast, White men show higher TPR across education levels:

- **White men, less than high school:** TPR=24.1%
- **White men, high school:** TPR=35.3%
- **White men, some college:** TPR=15.4%
- **White men, college+:** TPR=36.1%

These patterns demonstrate that educational attainment does not fully mitigate race/sex-based biomarker performance gaps, consistent with intersectionality theory and research on diminishing returns to socioeconomic mobility for Black women [58].

Supplementary Discussion

Comparison to ADNI and Other Clinical Cohorts

Our HRS sample differs markedly from ADNI participants along multiple dimensions (Table S9). ADNI is predominantly White (>90%), highly educated (>60% college+), and older (mean age 75 vs. 68.3 in HRS). These demographic differences have profound implications for biomarker performance estimates.

ADNI-derived cutpoints and effect sizes may not transport to general populations. For example, ADNI reports amyloid-cognition associations of $\beta \approx -1.0$ [32], substantially larger than our weighted estimate ($\beta = 0.11$, non-significant). This discrepancy likely reflects ADNI's enrichment for "pure" AD pathology, higher baseline cognition, and lower comorbidity burden compared to HRS.

Population-representative cohorts such as the HRS provide essential complementary evidence to clinic-based studies, enabling evaluation of real-world performance and identification of equity gaps invisible in homogeneous convenience samples.

Implications for Blood-Based Screening Programs

Proposed blood-based AD screening programs [4] must grapple with the equity gaps documented here. If implemented with current biomarkers and universal cutpoints, such programs would systematically under-identify at-risk individuals in Black, Hispanic, and low-education populations due to lower TPR.

This differential sensitivity could exacerbate health disparities by channeling resources (confirmatory testing, early intervention, clinical trials) preferentially to already-advantaged groups. Equity-conscious implementation would require:

1. **Subgroup-specific cutpoints** calibrated to equalize TPR across demographics
2. **Multi-biomarker algorithms** integrating vascular, inflammatory, and metabolic markers to

capture heterogeneous pathology
3. **Community engagement** to ensure acceptable and culturally appropriate implementation
4. **Longitudinal monitoring** for fairness drift and unintended consequences
5. **Resource allocation** targeting underserved populations for enhanced follow-up

Without these safeguards, blood-based screening risks becoming another tool that widens rather than narrows health disparities.

Ethical Considerations for Race-Specific Algorithms

Our race-specific cutpoint analysis (Table S7) raises profound ethical questions. While empirically justified by observed performance differences, race-specific algorithms risk[9]:

1. **Reifying biological race:** Treating race as a biological rather than social construct 2. **Masking structural causes:** Attributing disparities to inherent racial differences rather than racism and structural disadvantage 3. **Perpetuating stereotypes:** Reinforcing harmful beliefs about racial differences in cognition or brain health 4. **Enabling discrimination:** Providing tools that could be weaponized for exclusion or differential treatment

Alternative approaches merit consideration:

- **Continuous risk scores** without categorical cutpoints, calibrated within subgroups
- **Fairness-constrained algorithms** that optimize for equitable performance without explicit race encoding
- **Structural covariate inclusion** (education, neighborhood disadvantage, discrimination exposure) that captures social determinants without race labels
- **Patient-centered shared decision-making** that incorporates biomarkers as one data point among many, rather than deterministic thresholds

Any implementation must be guided by affected communities, monitored rigorously for harms, and subject to revision based on real-world equity impacts.

Role of Structural Racism in Observed Disparities

Our education-stratified analyses (Tables 7-9; Figures 4-5) demonstrate that structural disadvantage modifies biomarker-cognition relationships. However, education is an imperfect proxy for lifetime socioeconomic adversity and does not capture the full scope of structural racism's health impacts.

Structural racism operates through multiple pathways relevant to dementia risk:

- **Environmental exposures:** Residential segregation concentrates air pollution, lead, and other neurotoxins in minoritized neighborhoods [39]
- **Chronic stress:** Discrimination experiences activate inflammatory pathways and accelerate biological aging [58,59]
- **Healthcare access:** Systemic barriers limit preventive care, treatment quality, and clinical trial

participation [62]

- **Wealth inequality:** Intergenerational wealth gaps constrain resources for brain-healthy lifestyles [45]

These mechanisms are not captured by ATN biomarkers optimized for amyloid and tau pathology. Future work should integrate measures of structural racism (neighborhood disadvantage indices, discrimination scales, wealth assessments) to illuminate pathways linking social injustice to dementia disparities.

SUPPLEMENTARY TABLES

Table S1. Vascular Comorbidity Prevalence by Race/Ethnicity

Race/Ethnicity	N	Diabetes (%)	Hypertension (%)	Stroke (%)	Any CVD (%)	Mean CVD Burden (SD)
**Black**	738	35.7	78.2	10.0	82.1	1.24 (0.91)
**Hispanic**	661	38.1	64.7	5.9	73.1	1.09 (0.88)
**Other**	143	36.2	58.2	4.9	64.7	0.99 (0.86)
**White**	2,885	22.5	59.4	6.9	64.6	0.88 (0.84)

Note: CVD = cardiovascular disease. Any CVD is defined as the presence of one or more conditions (diabetes, hypertension, or stroke). CVD Burden is the sum of these three conditions (range 0-3). Black participants show the highest prevalence across all vascular conditions, with 82.1% having at least one comorbidity. This elevated vascular burden may contribute to cognitive impairment through mechanisms independent of Alzheimer's pathology, potentially confounding biomarker-cognition associations.

Table S2. Vascular Comorbidity Prevalence by Race × Sex

Race/Ethnicity	Sex	N	Diabetes (%)	Hypertension (%)	Any CVD (%)
**Black**	Male	249	34.1	79.8	83.7
**Black**	Female	489	36.6	77.4	81.3
**White**	Male	1,243	25.8	63.1	68.4
**White**	Female	1,642	20.0	56.6	61.8

Note: Table restricted to Black and White participants due to sample size considerations for intersectional analyses. Within racial groups, sex differences in vascular burden are modest (2-7 percentage points), while between-race differences are substantial (15-20 percentage points). Black men show the highest vascular burden (83.7% any CVD), while White women show the lowest (61.8%).

Table S3. Biomarker-Cognition Associations With and Without Vascular Adjustment

Model	Biomarker	β Coefficient	SE	95% CI	P Value
**Base (Demographics Only)**					
	Amyloid+	0.108	0.137	(-0.167, 0.384)	0.432

	Tau+	-0.740	0.188	(-1.119, -0.360)	<0.001
	Neurodegeneration+	-0.270	0.153	(-0.578, 0.037)	0.083
**Adjusted for Vascular Comorbidity**					
	Amyloid+	0.110	0.133	(-0.158, 0.379)	0.411
	Tau+	-0.713	0.195	(-1.107, -0.320)	<0.001
	Neurodegeneration+	-0.204	0.145	(-0.497, 0.089)	0.168

Note: Base model adjusts for age, sex, race/ethnicity, and education. The vascular-adjusted model additionally includes hypertension, diabetes, and stroke as covariates. Survey-weighted regression with complex sampling design (SECU, STRATUM, PVBSWGTR). Outcome is cognitive score (TICSm, range 0-27). Vascular adjustment produces modest attenuation for tau (4%) and neurodegeneration (26%), with no change for amyloid. Tau remains robustly significant after vascular adjustment, indicating AD pathology effects are partially independent of cerebrovascular burden.

Table S4. Biomarker-Cognition Associations Stratified by CVD Status

CVD Status	N	Biomarker	β Coefficient	SE	95% CI	P Value
**No CVD**	1,341	Amyloid+	0.034	0.207	(-0.371, 0.440)	0.867
		Tau+	-1.082	0.296	(-1.663, -0.500)	<0.001
		Neurodegeneration+	-0.294	0.271	(-0.825, 0.237)	0.277
**Any CVD**	2,955	Amyloid+	0.273	0.140	(-0.001, 0.547)	0.051
		Tau+	-0.721	0.176	(-1.066, -0.376)	<0.001
		Neurodegeneration+	-0.515	0.177	(-0.861, -0.168)	0.004

Note: CVD = cardiovascular disease, defined as the presence of any of the following: hypertension, diabetes, or stroke. Models adjust for age, sex, race/ethnicity, and education. Outcome is cognitive score (TICSm). Biomarker associations persist within both CVD-free and CVD-present subgroups. Tau shows robust associations regardless of vascular burden, while neurodegeneration shows stronger effects in the CVD-present group, potentially reflecting combined AD and vascular contributions to brain injury. The larger tau effect in CVD-free individuals (β=-1.08) suggests particularly strong AD-specific associations in those without competing vascular pathology.

Table S5. Calibration Data by Race and Risk Decile

Selected rows for illustration; full table available in supplementary data file

Race/Ethnicity	Risk Decile	N	Predicted Risk	Observed Rate
**White**	1	263	0.114	0.080
**White**	5	293	0.173	0.109
**White**	10	329	0.360	0.322
**Black**	1	103	0.114	0.194
**Black**	5	71	0.166	0.282
**Black**	10	61	0.361	0.574
**Hispanic**	1	67	0.114	0.313
**Hispanic**	5	66	0.154	0.273
**Hispanic**	10	44	0.362	0.614

Note: Predicted risk derived from logistic regression with A+T+N biomarkers predicting cognitive impairment (CIND or dementia). Participants were divided into deciles of predicted risk within each racial/ethnic group. Perfect calibration corresponds to Predicted Risk = Observed Rate. White participants show reasonable calibration with predicted and observed rates tracking closely. Black and Hispanic participants show systematic miscalibration: underprediction at low risk (observed > predicted) and overprediction at high risk in some deciles, indicating models developed in predominantly White samples do not transport accurately.

Table S6. Calibration Statistics by Race/Ethnicity

Race/Ethnicity	N	Mean Predicted	Mean Observed	Calibration Slope	Calibration Intercept
**White**	2,885	0.211	0.151	0.988	-0.057
**Black**	738	0.191	0.308	1.292	0.060
**Hispanic**	661	0.185	0.307	1.777	-0.022
**Other**	143	0.186	0.231	1.652	-0.076

Note: Calibration slope and intercept derived from regressing observed cognitive impairment on predicted risk. Perfect calibration: slope = 1.0, intercept = 0.0. Slope > 1 indicates overfitting (predictions too extreme); slope < 1 indicates underfitting (predictions too conservative). Intercept < 0 indicates

systematic overprediction; intercept > 0 indicates underprediction. White participants show near-perfect calibration (slope=0.99, intercept=-0.06), while Black and Hispanic participants show poor calibration with slopes >1.2, indicating that risk models developed in predominantly White samples systematically misclassify minoritized individuals.

Table S7. Youden-Optimized Biomarker Cutpoints by Race/Ethnicity

Race	Biomarker	Optimal Cutpoint	Youden Index	AUC	Sensitivity	Specificity
**Overall**	Aβ42/40	0.077	0.049	0.518	0.193	0.856
	pTau181	1.989 pg/mL	0.201	0.612	0.501	0.700
	NfL	24.4 pg/mL	0.236	0.647	0.499	0.737
	GFAP	102.6 pg/mL	0.217	0.631	0.550	0.667
**White**	Aβ42/40	0.057	0.069	0.525	0.714	0.355
	pTau181	2.160 pg/mL	0.261	0.655	0.560	0.702
	NfL	24.0 pg/mL	0.298	0.696	0.614	0.684
	GFAP	102.9 pg/mL	0.281	0.671	0.652	0.629
**Black**	Aβ42/40	0.071	0.147	0.577	0.443	0.704
	pTau181	1.539 pg/mL	0.217	0.609	0.573	0.644
	NfL	17.6 pg/mL	0.261	0.645	0.557	0.704
	GFAP	119.5 pg/mL	0.207	0.617	0.417	0.790
**Hispanic**	Aβ42/40	0.076	0.084	0.525	0.260	0.824
	pTau181	1.737 pg/mL	0.226	0.634	0.446	0.780
	NfL	21.6 pg/mL	0.297	0.666	0.493	0.804
	GFAP	89.0 pg/mL	0.235	0.638	0.498	0.737

Note: Optimal cutpoints derived using Youden index maximization (Sensitivity + Specificity – 1) within each racial/ethnic group separately. Outcome is cognitive impairment (CIND or dementia). Substantial differences emerge across groups: pTau181 optimal cutpoint varies from 1.54 pg/mL (Black) to 2.16 pg/mL (White), a 40% relative difference. NfL ranges from 17.6 pg/mL (Black) to 24.0 pg/mL (White), a 36% difference. These race-specific optima provide empirical justification for considering population-specific thresholds, though ethical concerns about reifying biological race concepts must be carefully weighed against potential equity benefits.

Table S8. Fairness Landscape Across Race × Sex × Education

Race/Ethnicity	Sex	Education	N	TP	FN	TPR	N Impaired
Black	Male	Less than HS	59	6	22	0.2143	28
Black	Male	HS graduate	89	7	34	0.1707	41
Black	Male	Some college	52	0	16	0.0000	16
Black	Male	College+	52	1	6	0.1429	7
Black	Female	Less than HS	112	5	58	0.0794	63
Black	Female	HS graduate	143	4	38	0.0952	42
Black	Female	Some college	143	1	18	0.0526	19
Black	Female	College+	92	2	10	0.1667	12
White	Male	Less than HS	129	13	41	0.2407	54
White	Male	HS graduate	383	30	55	0.3529	85
White	Male	Some college	303	6	33	0.1538	39
White	Male	College+	430	13	23	0.3611	36
White	Female	Less than HS	180	13	43	0.2321	56
White	Female	HS graduate	563	12	84	0.1250	96
White	Female	Some college	456	16	36	0.3077	52
White	Female	College+	451	0	22	0.0000	22

Note: True positive rates (TPR) vary widely across intersectional groups, revealing substantial disparities in algorithmic sensitivity. Black women, especially those with lower educational attainment, exhibit the lowest TPRs despite comparable impairment counts, suggesting systematic under-identification. In contrast, White men with higher education show the highest sensitivity. These patterns demonstrate how race, sex, and education jointly shape model performance, emphasizing the need for fairness-aware calibration and validation. Small shifts in sensitivity across subgroups can meaningfully influence who is flagged for follow-up evaluation, with downstream implications for diagnostic equity, clinical trial access, and population-level burden estimates.

Table S9. HRS vs. ADNI Cohort Comparison

Characteristic	HRS (This Study)	ADNI (Shaw 2009)
**Sample Size**	4,427	416

**Mean Age (years)**	68.3	75.0
**% Female**	59.2	~45*
**% White**	65.2 (unweighted) / 78.9 (weighted)	>90*
**% College+**	25.8 (unweighted) / 31.0 (weighted)	>60*
**Sampling Frame**	Population-representative	Clinic-based volunteers
**Survey Weights**	Yes	No
**Geographic Coverage**	U.S. national	Select academic centers

Note: ADNI values marked with * are approximate based on published reports [87]. HRS provides population-representative estimates through probability sampling and survey weighting, while ADNI uses convenience sampling from academic medical centers. These demographic differences have profound implications for biomarker performance estimates. ADNI's enrichment for highly educated, predominantly White participants and “pure” AD pathology likely inflates biomarker-cognition effect sizes compared to HRS's more diverse, comorbidity-rich population. This comparison underscores the complementary value of population-representative cohorts for evaluating real-world biomarker performance and identifying equity gaps invisible in homogeneous convenience samples.

SUPPLEMENTARY FIGURES

Figure S1. Impact of Vascular Comorbidity Adjustment on Biomarker-Cognition Associations

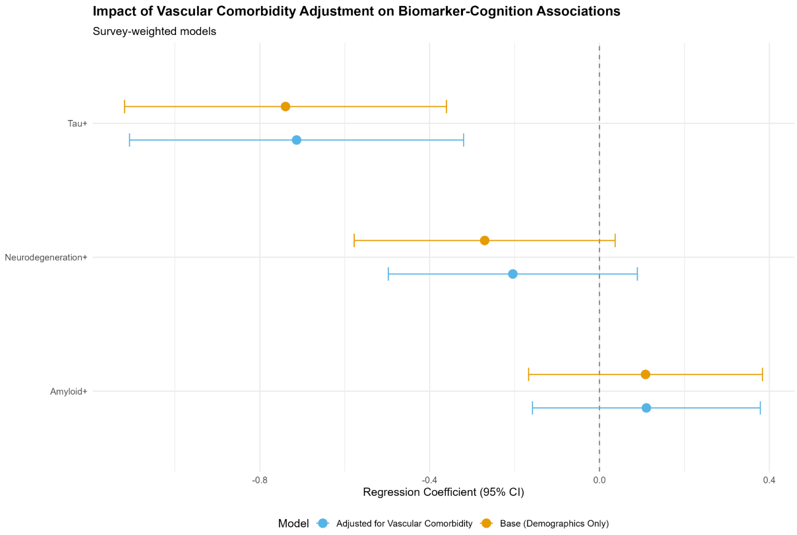

Note: Forest plot showing regression coefficients and 95% confidence intervals for biomarker-cognition associations. Two models per biomarker (amyloid, tau, neurodegeneration): Base model (demographics only) in orange, Vascular-adjusted model (demographics + hypertension + diabetes + stroke) in blue. Vertical dashed line at $\beta=0$ indicating null effect. X-axis: Regression Coefficient (95% CI). Y-axis: Biomarker type.

Figure S1 Legend: Forest plot comparing biomarker-cognition associations before and after adjustment for vascular comorbidities (hypertension, diabetes, stroke). Base model (orange) adjusts for age, sex, race/ethnicity, and education. Vascular-adjusted model (blue) additionally includes cardiovascular disease indicators. Survey-weighted regression coefficients with 95% confidence intervals shown. Outcome is cognitive score (TICSm, range 0-27). Tau shows minimal attenuation (4%) after vascular adjustment and remains highly significant ($p<0.001$), indicating AD pathology effects are partially independent of cerebrovascular burden. Neurodegeneration shows 26% attenuation, potentially reflecting combined AD and vascular contributions to NfL/GFAP elevations. Amyloid shows no change, remaining non-significant in both models. These patterns support the hypothesis that plasma tau specifically captures AD-related cognitive decline, while neurodegeneration markers may reflect more heterogeneous brain injury mechanisms.

Figure S2. CVD-Stratified Biomarker-Cognition Associations

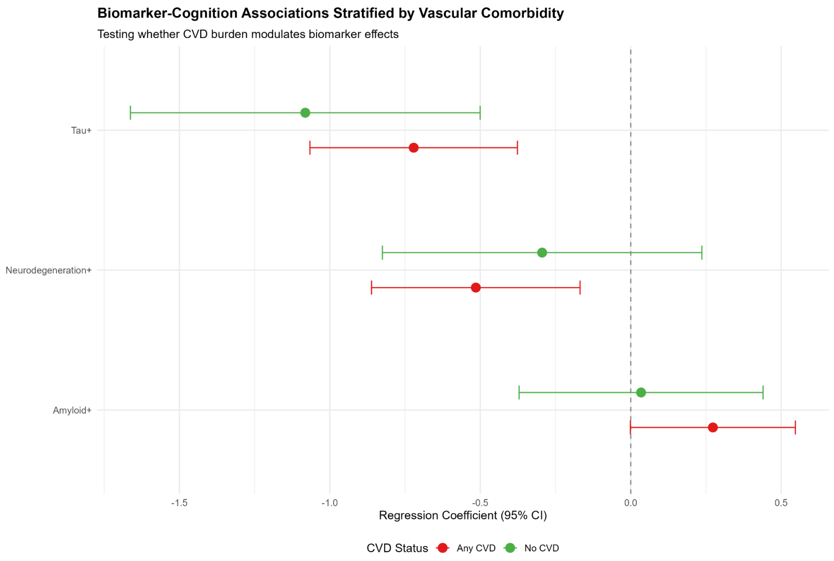

Note: Forest plot showing regression coefficients and 95% confidence intervals for biomarker-cognition associations stratified by CVD status. Two groups per biomarker (amyloid, tau, neurodegeneration): No CVD (n=1,341) in green, Any CVD (n=2,955) in red. Vertical dashed line at β=0 indicating null effect. **X-axis:** Regression Coefficient (95% CI). **Y-axis:** Biomarker type.

Figure S2 Legend: Forest plot displaying biomarker-cognition associations stratified by cardiovascular disease (CVD) presence. No CVD (green) includes participants without hypertension, diabetes, or stroke. Any CVD (red) includes participants with one or more vascular conditions. All models adjust for age, sex, race/ethnicity, and education. Outcome is cognitive score (TICSm). Tau demonstrates robust associations in both strata, with particularly strong effects in CVD-free individuals (β=-1.08, $p<0.001$), suggesting powerful AD-specific associations when competing vascular pathology is absent. Neurodegeneration shows significant associations only in the Any CVD stratum (β=-0.52, p=0.004), consistent with NfL/GFAP capturing both AD and vascular brain injury. Amyloid remains non-significant in both strata. These findings indicate that biomarker-cognition relationships persist independent of vascular burden, though effect magnitudes vary, supporting partially distinct AD and vascular pathways to cognitive decline.

Figure S3. Biomarker Calibration by Race/Ethnicity

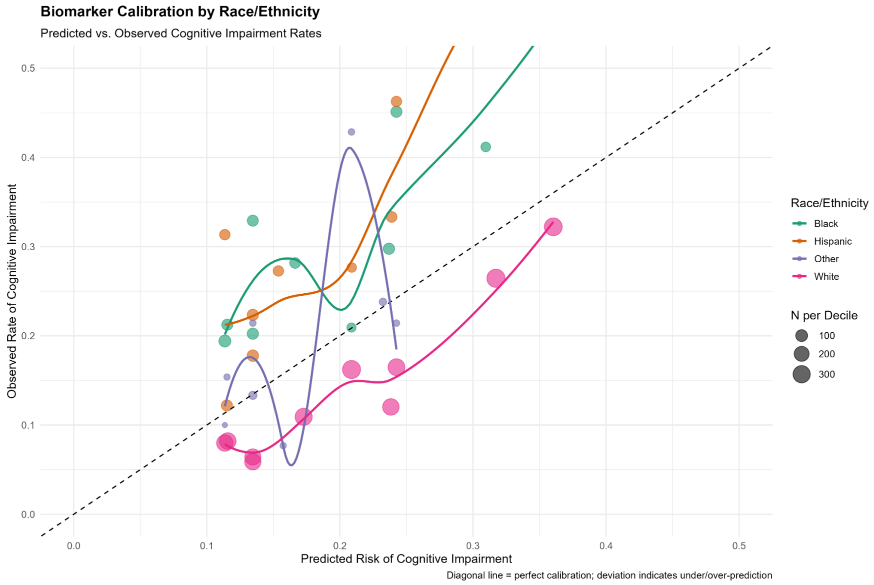

Note: Calibration plot with predicted risk of cognitive impairment (X-axis, 0-0.5) vs. observed rate of cognitive impairment (Y-axis, 0-0.5). Points represent risk deciles within each racial/ethnic group, sized by sample size, colored by race/ethnicity (White=pink, Black=teal, Hispanic=orange, Other=purple). Smooth loess curves fitted for each group. Diagonal dashed line indicating perfect calibration (predicted = observed). Points on the diagonal indicate good calibration; points above the diagonal indicate underprediction; points below the diagonal indicate overprediction.

Figure S3 Legend: Calibration plot comparing predicted vs. observed cognitive impairment rates across risk deciles, stratified by race/ethnicity. Predicted risk derived from logistic regression, including A+T+N biomarkers as predictors. Outcome is cognitive impairment (CIND or dementia). Each point represents a risk decile within a racial/ethnic group, with point size proportional to sample size (N per decile). Perfect calibration corresponds to points lying on the 45-degree diagonal (dashed black line); deviation indicates systematic under- or over-prediction. White participants (pink) show reasonable calibration with points clustering near the diagonal. Black (teal) and Hispanic (orange) participants show systematic miscalibration: observed rates exceed predicted rates at low risk (underprediction) and deviate unpredictably at high risk. Loess smoothing curves illustrate group-specific calibration patterns. These calibration failures indicate that biomarker-based risk models developed in predominantly White samples do not transport accurately to minoritized populations, necessitating race-specific recalibration or alternative modeling approaches to achieve equitable performance.

Figure S4. Race-Specific vs. Universal Optimal Biomarker Cutpoints

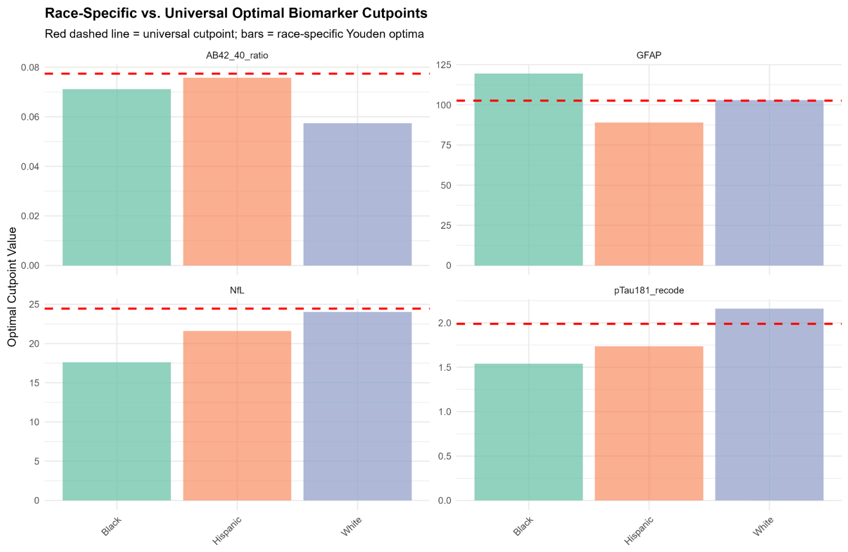

Note: Grouped bar chart with biomarkers (Aβ42/40, pTau181, NfL, GFAP) on X-axis, optimal cutpoint value on Y-axis. Bars colored by race/ethnicity (Black=teal, Hispanic=orange, White=purple). Horizontal red dashed lines indicate the universal (overall) optimal cutpoint for each biomarker. Faceted by biomarker with free Y-axis scales to accommodate different measurement units.

Figure S4 Legend: Comparison of race-specific Youden-optimized biomarker cutpoints vs. universal (overall population) thresholds. Bars represent optimal cutpoints for each racial/ethnic group derived by maximizing the Youden index (Sensitivity + Specificity – 1) within the group. Red dashed horizontal lines indicate universal cutpoints optimized across the entire sample. Substantial differences emerge: pTau181 optimal cutpoint varies from 1.54 pg/mL (Black) to 2.16 pg/mL (White), a 40% relative difference. NfL ranges from 17.6 pg/mL (Black) to 24.0 pg/mL (White), a 36% difference. GFAP shows a reversed pattern, with Black participants having a higher optimal threshold (119.5 pg/mL) than Hispanic (89.0 pg/mL) or White (102.9 pg/mL) participants. These empirically-derived race-specific optima suggest that universal cutpoints may systematically misclassify minoritized individuals, contributing to observed fairness disparities (12-percentage-point TPR gap). However, implementing race-specific thresholds raises ethical concerns about reifying biological race concepts and requires careful consideration of potential benefits vs. harms.

Figure S5. Fairness Landscape: TPR Across Race × Sex × Education

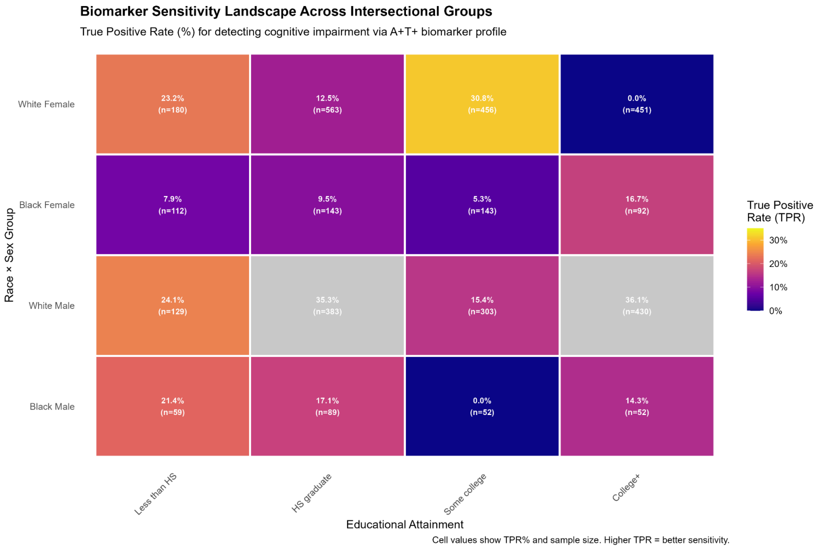

Note: Heatmap with Education level (Less than HS, HS graduate, Some college, College+) on X-axis and Race × Sex Group (White Female, Black Female, White Male, Black Male) on Y-axis. Cells colored by True Positive Rate (TPR) using plasma color scale (dark blue=0%, bright yellow=35%). Cell values display “TPR% (n=sample size)”. Higher TPR indicates better biomarker sensitivity for detecting cognitive impairment.

Figure S5 Legend: Heatmap visualizing biomarker sensitivity landscape across intersectional demographic groups defined by race, sex, and educational attainment. True Positive Rate (TPR) represents the proportion of individuals with cognitive impairment correctly identified by the A+T+ biomarker profile. Color intensity indicates TPR magnitude (dark blue=low sensitivity, bright yellow=high sensitivity). Cell values show TPR percentage and sample size. Black women exhibit persistently low TPR across all education levels (range 0.0-16.7%), with particularly striking deficits in higher education strata (some college: 5.3%, college+: 16.7%). White men show the highest TPR across education levels (range 15.4-36.1%). These patterns demonstrate that educational attainment does not fully mitigate race/sex-based biomarker performance gaps, consistent with intersectionality theory positing that multiple marginalized identities produce unique forms of disadvantage not reducible to additive effects. The finding that Black women with a college education show TPR=16.7% (vs. White men with less than HS showing TPR=24.1%) illustrates how racism and sexism compound to produce systematic biomarker performance disparities even among highly educated minoritized individuals.
